# Genome sequencing and analysis of fungus *Hirsutella sinensis* isolated from *Ophiocordyceps sinensis*

**DOI:** 10.1186/s13568-020-01039-x

**Published:** 2020-06-03

**Authors:** Li-Qun Jin, Zhe-Wen Xu, Bo Zhang, Ming Yi, Chun-Yue Weng, Shan Lin, Hui Wu, Xiang-Tian Qin, Feng Xu, Yi Teng, Shui-Jin Yuan, Zhi-Qiang Liu, Yu-Guo Zheng

**Affiliations:** 1 Key Laboratory of Bioorganic Synthesis of Zhejiang Province, College of Biotechnology and Bioengineering, Zhejiang University of Technology, Hangzhou, 310014 China; 2 HuaDong Medicine (Hangzhou) Bailing Biological Technology Co., Ltd, Hangzhou, 311220 China; 3 East China Pharmaceutical Group Limited Co., Ltd, Hangzhou, 311000 China

**Keywords:** *Hirsutella sinensis*, Genome sequencing, Biosynthesis pathways of ingredients, Traditional Chinese medicine

## Abstract

*Ophiocordyceps sinensis* has been used as a traditional medicine or healthy food in China for thousands of years. *Hirsutella sinensis* was reported as the only correct anamorph of *O. sinensis*. It is reported that the laboratory-grown *H. sinensis* mycelium has similar clinical efficacy and less associated toxicity compared to the wild *O. sinensis*. The research of the *H. sinensis* is becoming more and more important and urgent. To gain deeper insight into the biological and pharmacological mechanisms, we sequenced the genome of *H. sinensis*. The genome of *H. sinensis* (102.72 Mb) was obtained for the first time, with > 99% coverage. 10,200 protein-encoding genes were predicted based on the genome sequence. A detailed secondary metabolism analysis and structure verification of the main ingredients were performed, and the biosynthesis pathways of seven ingredients (mannitol, cordycepin, purine nucleotides, pyrimidine nucleotides, unsaturated fatty acid, cordyceps polysaccharide and sphingolipid) were predicted and drawn. Furthermore, infection process and mechanism of *H. sinensis* were studied and elaborated in this article. The enzymes involved in the infection mechanism were also predicted, cloned and expressed to verify the mechanism. The genes and proteins were predicted and annotated based on the genome sequence. The pathways of several active components in *H. sinensis* were predicted and key enzymes were confirmed. The work presented here would improve the understanding of the genetic basis of this organism, and contribute to further research, production and application of *H. sinensis*.

## Introduction

*Ophiocordyceps sinensis*, a fungus that parasitizes *Lepidoptera* larvae, has been used as a traditional medicine or healthy food in China for thousands of years (Zhang et al. 2012; Zhu et al. 1998). The use of *O. sinensis* has a long history in traditional Chinese medicine and Tibetan medicine (Sharma 2004; Winkler 2008). It has been reported that *H. sinensis* is the only correct anamorph of *O. sinensis*. There are high function similarities between wild *O. sinensis* and cultured *H. sinensis*,both of them be used to treat weakness after sickness, lung and kidney-associated diseases and sexual dysfunction (Buenz et al. 2005; Chen et al. 2001; Li et al. 2006; Zhou et al. 2009). Recently, it is found that *H. sinensis* has activities to modulate immune responses, inhibit tumor cell proliferation, enhance hepatic function, regulate insulin sensitivity, decrease plasma cholesterol levels and modulate steroidogenesis (Cheung et al. 2009; Fu et al. 2018; He et al. 2013; Lu et al. 2019).

For thousands of years, *O. sinensis* can only be obtained by field collection, so wild *O. sinensis* was called as ‘golden Chinese traditional medicine’ (Chen et al. 2017). In recent years, due to high demand and the excessive excavation, the supply of *O. sinensis* is almost exhausted in many areas (Huang et al. 2017). Because of its important applications in the Chinese traditional medicine, it is increasingly urgent to carry out the genetic studies of *O. sinensis* to discover valuable secondary metabolic functional genes, elucidate related metabolic pathways and to pave foundation for development of new approaches for pharmacological utilization. Investigation of *O. sinensis* in molecular and genetic levels also would provide new insight into this organism and aid in its development and retention (Zhang et al. 2018).

Recently, *H. sinensis* mycelia have been reported to have similar clinical efficacy and less associated toxicity compared with wild *O. sinensis*, and *H. sinensis* is expected to be an alternative of *O. sinensis* (Liu et al. 2015). The main biological and pharmacological active ingredients in *H. sinensis* are mannitol, cordycepin, purine nucleotides, pyrimidine nucleotides, unsaturated fatty acid, cordyceps polysaccharide and sphingolipid, which are consistent with the active ingredient in wild *O. sinensis* (Du et al. 2017; Olatunji et al. 2018). Thus, artificially cultured mycelia of *H. sinensis* have become increasingly used in medicinal products (Li et al. 2019).

In order to clarify the biological and pharmacological mechanisms of *O. sinensis*, we carried out the genome sequencing of *H. sinensis,* anamorph of *O. sinensis*, for the first time, described the annotation and gene expression, and analyzed the complete sequencing data (Li et al. 2016). Genome content, evolutionary analyses, and investigation of some of the genetic features underlying the unique biology of *H. sinensis* have been defined according to the analysis from complete sequence data. Then genes and enzymes which control the biosynthesis of the active ingredients were obtained according to the sequencing and annotation results. We further investigated the interaction of different pathways and corresponding enzymes, and tried to find out the real medicinal ingredients and how they work. Also, structure verification of the main ingredients was employed to clarify. The infection mechanism was investigated to reveal the microbial properties of *H. sinensis* (Olatunji et al. 2018). The genome sequence obtained in this study will facilitate understanding of the genetic basis of many traits at genome level and allow the undertaking of genome-wide association studies of *H. sinensis* (Tang et al. 2015). Several features of *H. sinensis* were investigated based on its genome. The availability of genome will also facilitate the identification and manipulation of candidate genes or genomic regions to generate the new ways to synthesize new compounds with potentials in pharmaceuticals, and pave foundation for development of new drugs for the pharmaceutical manufacturing or provide the theoretical basis for the realization of sustainable resource use of *H. sinensis*; improve the research of *H. sinensis* and obtain a more efficient processes of production. The work present here would aid in understanding and carrying out future research on the genetic basis of biology of this organism and contribute to the further production and application of *H. sinensis*. In this way, it would contribute to the world-wide application of *O. sinensis*.

## Materials and methods

### Isolation and identification of *H. sinensis*

The *O. sinensis* samples were collected at the surface and various depths at different locations in Yushu, Qinghai province. The temperature on the sampling sites was 28–34 °C in dry seasons and 11–17 °C in wet seasons. Samples were cultured at least 16 h after collection and collected in sterile plastic containers. Subsequently, the samples were cultured in saline, and then transported to the laboratory by sterilized poly-ethylene bags.

In order to isolate *H. sinensis*, several isolation procedures were carried out. Fresh and complete *O. sinensis* were first selected out, and impurities on the surface of fruiting bodies were cleaned up by sterile water. Then fruiting bodies were washed several times with sterile purified water, and disinfection was carried out by conventional method by using 0.1% mercuric chloride. Subsequently, worms and stromata were carefully separated by sterile scalpel in sterile conditions. Three parts of the tissues were picked and cultured on the sterilized PDA slant medium at 16 °C. Growth condition was recorded daily. In addition, worms and stromata were broken apart with sterile forceps, and center white mycelium tissues were seeded in PDA medium.

The isolation media of potato dextrose agar (PDA; Difco, Detroit, MI, USA) was prepared and then autoclaved at 115 °C for 30 min before use. Liquid PDA medium was composed 20% potatoes, 2.0 g/L glucose, 0.46 g/L KH_2_PO_4_, 0.5 g/L MgSO_4_·H_2_O, 10.0 mg/L VB_1_, 1.0 mg/L K_2_HPO_4_. Solid PDA medium needs another 2% agar. The fermentation medium: 1.0% glucose, 1.0% molasses, 0.5% silkworm chrysalis powder, 1.0% soybean meal, 0.5% yeast extract, 0.01% MgSO_4_·H_2_O, 0.02% KH_2_PO_4_.

### Genome sequencing and analysis

High-quality genomic DNA was prepared from *H. sinensis* using a standard phenol/chloroform method. To prevent RNA and protein contamination, the prepared DNA was treated with RNase A and proteinase K. The detailed procedure of phenol/chloroform method was described as follows: 0.5 g ground powder of *H. sinensis* was resuspended in 1 mL TE buffer containing 110 μL 10% SDS, 4.2 μL 20 mg/mL proteinase K, and they were mixed thoroughly. The solution was incubated at 50 °C for 2 h. After incubation, the solution was further centrifuged at 10,000*g* for 15 min, and the supernatant was transferred into a fresh 1.5 mL tube. The same volume of phenol/chloroform/isoamylol (25/24/1) was added, and mixed gently and thoroughly. The mixture was centrifuged at 12,000*g* for 10 min, the supernatant was transferred into a 1.5 mL ep tube, and the same volume of chloroform/isoamylol (24/1) was added. The solution was mixed thoroughly and centrifuged at 12,000*g* for 10 min. The supernatant was transferred into a new 1.5 mL ep tube, and the same volume of isopropanol was added. The mixture was incubated at 25 °C for 30 min until sediment formed. The sediment was transferred into 75% ethanol solution and rinsed two times. After centrifuged at 12,000*g* for 15 min, the sediment was dried at room temperature, and dissolved into 60 μL TE buffer.

Using genomic DNA as a template, PCR amplification was carried out using universal primers (BioRad Corporation of America, PTC200 Amplifier). The reaction conditions were: pre-denaturation at 95 °C for 5 min, cycle parameters were denatured at 94 °C for 45 s, and renatured at 55 °C for 60 s. After 72 °C extension for 90 s, after 35 cycles, 72 °C extended for 10 min.

Blast sequence alignment was performed on NCBI’s Genbank (http://blast.ncbi.nlm.nih.gov/). The sequences of ITS regions in several Genbanks with the obtained sequences in one order, family, and genus were selected, multiple sequence alignment was performed using CLUSTAL, and the evolutionary tree evaluation of these sequences was performed using Bootstrap method.

### Whole genome sequencing and assembly

Illumina Genome Analyzer Sequencing technology and Hiseq 2000 Sequencing System (Illumina, Inc., San Diego, CA, USA) were used to perform the whole genome shot-gun sequencing. We constructed 16 sequencing libraries with insert sizes of about 200 bp, 350 bp, 500 bp, 1 kb, 2 kb, 5 kb, 10 kb, and 20 kb. After genome sequencing, a series of checking and filtering steps on generated reads were performed to reduce the effect of sequencing error on the assembly. 28.97 Gb clean data were used for de novo genome assembly. SOAPdenovo software (BIG, Shenzhen, China) was utilized to carry out the whole genome assembly. The contigs without any gaps were obtained after SOAPdenovo assembly and correction. Subsequently, the obtained reads were aligned with contig sequences and paired ends. Meanwhile, the relationship and consistency between each pair of contigs were evaluated, and scaffolds were constructed step by step. Thereafter, 86,812 Mb raw data were obtained from 18 sequencing libraries of *H. sinensis.* Subsequently, in order to obtain super scaffolds, we used 29,662.8 Mb clean data for mapping to scaffolds. Finally, the genetic map was used to comprise marker loci. To narrow the gap inside the constructed scaffolds, which were primarily composed of masked repeats before scaffold construction, the paired end information was used to retrieve the read pairs that had one end mapped to the unique contig and the other located in the gap region. Furthermore, local assembly was performed for these collected reads.

In addition, we used Megablast (E-value < 1e−5, > 90% identity, > 200 bp length mapped to scaffold sequence) to check the quality of genome assembly for microbial contamination by alignment against a database of bacterial genomes.

### Identification of noncoding RNA genes

We searched the rRNA by comparison of the references, or used rRNAmmer software to predict rRNA and tRNA regions. The secondary structures of RNA were predicted by tRNAscan software and then sRNA was predicted by Rfam software. We predicted the tRNA genes using tRNAscan-SE with eukaryote parameters. The rRNA template sequences from *H. sinensis* were aligned by BlastN with E-value 1e^−5^ to identify the rRNA fragments. And we predicted the miRNA and snRNA genes using INFERNAL software against the Rfam database (Release 9.1).

In order to determine the rDNA loci cytogenetically in the *H. sinensis* genome, 18S rRNA gene and 5S rRNA gene were cloned for fluorescence in situ hybridization (FISH). We used nick translation method to directly label 18S rRNA gene with Texas red-12-dUTP, and 5S rRNA gene with Fluorescein-12-dUTP, respectively. Chromosomes were counterstained with 4, 6-diamidino-2-phenylindoleand (DAPI). We took images using Zeiss Axio Imager M2 microscope, which were equipped with AxioCamMRm and controlled by Axio Vision software. Finally, we adjusted the image for publication by Adobe Photoshop CS3 (Adobe Systems Inc, San Jose, CA, USA).

### Identification of repetitive elements

Tandem Repeats Finder was used to search the genome for tandem repeats, and Repbase (composed of many transposable elements) was also used to identify the interspersed repeats. We identified the transposable elements in the genome assembly at the DNA and protein levels, respectively. At the DNA level, a custom library comprising a combination of Repbase and the de novo transposable element library of the *H. sinensis* genome was used with the help of RepeatMasker. Subsequently, at the protein level, we performed RM-BlastX against the transposable element protein database by using RepeatProteinMask, which was updated in the RepeatMasker package. Under this background, the software RepeatModeler was used to build a new repeat library based on the genome. We used these results to construct a new library in the context that RepeatMasker and RepeatProteinMasker were carried out again to find homolog repeats in the genome. Furthermore, we classified the identified repeats into different classes as per standard genome analysis.

### Gene prediction

Gene sequences are obtained from assembly result using Augustus or other software. *CEGMA* software (Parra and Korf 2007) is based on the conserved genes in eukaryotes and was used to predict the core gene sequence. The repeat sequence is masked with N to reduce its effect on de novo prediction. We use *SNAP* and Augustus software separately to process de novo prediction with the training set obtained by the genes predicted by *CEGMA* software, which are relatively complete. We predicted genes by *Homology* with several Homologous species. Then, we merged the de novo and *Homology* predictions and the assembly sequence together with glean software to obtain the primary prediction result.

### Genome annotation and analysis

Three main approaches: homology-based method (H), de novo method (D) and EST/unigenes-based method (C) were used to predict genes. The results were first integrated and filtered by GLEAN program, and later examined again manually.

We performed prediction by using the protein sequences from five sequenced species, namely *Cordyceps militaris*, *Metarhizium anisopliae*, *Metarhizium acridum*, *O. sinensis*, and *Hirsutella minnesotensis*, on the condition of taking one species each time. TblastN with E-value 1e^−5^ was used to map them to the genome assembly. Thereafter, we aligned homologous genome sequences against matched proteins with the help of GeneWise (version 2.0) for accurate spliced alignments. Then, pseudogenes from the homology-search results from five data sets were filtered.

In term of de novo method, we applied Augustus, GENSCAN and GlimmerHMM to predict genes with parameters trained on *H. sinensis*. Three de novo predictions were merged into a unigene set. We retained the de novo gene models which were supported by two or more de novo methods. To overlap gene models, we selected the longest one and then got de novo-based gene models. In the third approach, the transcribed sequences and overlaps were used to link the spliced alignments using PASA (http://www.lerner.ccf.org/moleccard/qin/pasa/).

We distributed gene functions based on the best match of the alignments using BLASTP (1e^−5^) to SwissProt and TrEMBL databases. Motifs and domains of gene products were determined by InterProScan against protein databases including Pfam, PRINTS, PROSITE, ProDom and SMART. We obtained Gene Ontology IDs from the corresponding InterPro entries for each gene. We aligned all genes against KEGG proteins, and derived the pathway for the matched genes.

The corresponding functional annotation information can be obtained by comparing the gene sequences with the databases. The functional annotation is accomplished by analysis of protein sequences. We aligned genes to databases and obtained corresponding functional annotation. To ensure the biological functions, we choosen the highest quality alignment result as the annotation index to the genes. We used BLAST to accomplish all functional annotations by combination of different databases. We provided BLAST result in M8 format and the collection of annotation results was obtained through alignment with selected databases. Currently, we provided the alignment results through alignment with following databases: KEGG, COG, SwissProt, TrEMBL, NR and GO.

### Genome duplication estimation

The Vmatch software package (Stefan Kurtz, University of Hamburg, Germany) was used to generate clusters of similar genes based on sequence similarity in the predicted gene models. We further analyzed the resulting clusters, which were composed of two to six genes each, to determine duplicate gene pairs within each cluster using the yn00 program of PAML. Then, i-ADHoRe, which finds synthetic blocks by identifying successive pairs of duplicated genes, was used to analyze these duplicate pairs. We identified the first and last genes of each block, and recorded their positions in the genome in the GFF file, so that we could obtain the Circos image. To show the relative block size, we used Ribbon option in Circos to draw thick lines at the start and end points, which have a thickness that directly corresponds to the size of the duplicated block.

### Identification of ORFan genes

We identified the ORFans in the *H. sinensis* genome by a BLAST filtering approach (BLASTP, e-value < 0.01), and compared all predicted peptide sequences to all available peptide sequences in fully annotated *H. sinensis* genome. Peptides with the significant hit to a non-*H. sinensis* peptide were filtered out. Then, we searched the remaining *H. sinensis* ORFan candidates against the NCBI non-redundant protein and EST databases by BLASTP and t-BLASTN, respectively (e-value < 0.01). For the NCBI multi-species databases, we retrieved the species names of all significant hits by the gene accession and blastdb cmd program, and filtered out those *H. sinensis* peptides with hits to non-*H. sinensis* peptides again. In addition, we did further filtering by position-specific PSIBLAST based on the NCBI nr protein database (e-value < 0.01) and InterProScan. We only considered hits of type family for InterProScan, and then removed those ORFan candidates matching the taxonomic coverage family that extended past *H. sinensis.*

We identified ORFans originated by duplication events using all-against-all BLASTP and BLASTN searches for all ORFans versus non-ORFans within the *H. sinensis* genome, and identified orthologs containing frame shifts using BLASTN against all sequenced *H. sinensis* genome coding sequences. Furthermore, we also identified de novo origination and gene loss events by BLASTN against the genome assemblies of all sequenced *H. sinensis* genomes and compared the results to known open reading frames.

### Identification of SSRs and SNPs

We developed SSRs in the genome sequence by the MIcroSAtellite (MISA) program, under the following parameters: at least ten repeats for mono-, six repeats for di-, and five repeats for tri-, tetra-, penta- and hexa-nucleotide for simple SSRs. Subsequently, based on the following criteria: (i) annealing temperature (Tm) between 50 and 65 °C with 60 °C as optimum; (ii) product size ranging from 100 to 350 bp; (iii) primer length ranging from 18 to 24 bp with an optimum of 20 bp; (iv) GC  % content in the range of 40–60%, we used Primer5.0 program to design the primer pairs for identified SSRs.

SOAPdenovo (http://soap.genomics.org.cn/), which allows not more than two mismatches, was used to identify SNPs on the basis of alignment of Illumina transcript reads generated from each of the genotypes against the genome assembly. According to the alignment results, with consideration and analysis of data characters, sequence quality and other influences of experiments, we calculated the probability of genotypes with the actual data using the Bayesian model. We selected the genotype with the highest probability as the genotype of the sequenced individual at the specific locus and designated a quality value according to the reflected accuracy of the genotype. We selected the polymorphic loci against the reference sequence by the consensus sequence, and filtered them under certain requirements. Finally, we used two additional filter steps to remove unreliable portions of the consensus sequence: (i) the average copy times of all the reads mapped to this position would be less than twice. (ii) The SNPs had to be at least 3 bp away from each other.

### Estimating heterozygosity

Heterozygosity in *H. sinensis* was estimated in the following four steps. (i) The software SOAP2 (http://soap.geno mics.org.cn/soapaligner.htmL) with the cutoff of less than five mismatches was used to map all the high-quality reads from the genomic DNA of *H. sinensis* to the genome assembly. (ii) SOAPsnp (http://soap.genomics.org.cn/soapsnp.htmL) was used to analyze the read alignment results for SNP mining. (iii) Sites were searched and named as “criterion effective sites” according to the following criteria: (a) quality score of consensus genotype in the SNP mining result is more than 20; (b) count of all the best and second-best mapped bases are supported by at least four unique reads; (c) sequencing depth is greater than 10×; and (d) SNPs are at least 5 bp away from each other. (iv) in addition to the parameters for the criterion effective sites, the sites whose number of reads supported by the best base calling is less than four times, the number of reads supported by the second-best base calling (best base calling reads count < 4 s-best base calling reads count) were identified as heterozygosis sites. Ultimately, we estimated the rate of the heterozygosity as the number of heterozygosis sites divided by the number of criterion effective sites.

### Sequence alignment and phylogenetic analysis

Sequences were edited using SeqEd 1.0.3 (Applied Biosystems Inc., Carlsbad, CA, USA) and contigs were assembled using CodonCode Aligner 1.4 (CodonCode Inc., Centerville, MA, USA). Sequences of each gene partition were initially aligned by Clustal W 1.64 (Julie and Desmond 1994) and appended to an existing alignment (Wang et al. 2014). The initial alignment was manually edited if necessary in MacClade 4.0 (Maddison and Maddison 2000). All five gene regions sampled in this study were concatenated into a single, combined data set with ambiguously aligned regions excluded from phylogenetic analysis. Sequences of two additional gene regions, β-tubulin (tub) and mitochondrial ATP6 (atp6) were also combined with the data set to generate a supermatrix.

In order to detect incongruence among the five individual gene regions sampled in this study, bootstrap proportions were used for each individual data set with the taxa that was complete for all five genes. Bootstrap proportions (BP) were determined in a maximum-parsimony framework using the program PAUP 4.0b10. Only parsimony informative characters were used with the following search options: 100 replicates of random sequence addition, TBR branch swapping, and MulTrees OFF.

Maximum likelihood (ML) analyses were performed by RAxML-VI-HPC v2.2 using a GTRCAT model of evolution (Stamatakis et al. 2005). The model was separately applied to each of the 11 partitions, which consisted of nrSSU, nrLSU and the nine codon positions of 3 protein-coding genes (tef1, rpb1, and rpb2). Nodal support was assessed with nonparametric bootstrapping using 200 replicates. Bayesian Metropolis coupled Markov chain Monte Carlo (B-MCMCMC) analyses were performed on combined datasets using MrBayes 3.0b4 (Huelsenbeck and Ronquist 2001). In estimating the likelihood of each tree, we used the general time-reversible model, with invariant sites and gamma distribution (GTR + I + Γ) and employed the model separately for each partition. In an initial analysis, a B-MCMCMC analysis with five million generations and four chains were conducted in order to test the convergence of log-likelihood. Trees were sampled every 100 generations, to generate a total of 50,000 trees. For a second analysis, 5 independent Bayesian runs with two million generations and random starting trees were conducted to reconfirm the log-likelihood convergence and mixing of chains.

In addition to the analyses for the 162-taxon 5-gene data set, a series of analyses were conducted in MP, ML and Bayesian frameworks with different taxon samplings to address the potential topological effects of missing data. Previous phylogenetic and simulation studies demonstrated that the phylogenetic analyses are usually not negatively affected if fewer than 50% characters are missing for each taxon in the phylogenetic analyses (Philippe et al. 2004; Wiens 2003).

Although we have obtained the genome sequences of *H. sinensis*, the comparison among *H. sinensis* and other fungus species could not be performed for the reason that the similarity among their genomes is significantly low. ITS (internal transcribed spacer) refers to a piece of non-functional RNA situated between structural ribosomal RNAs (rRNA) in a common precursor transcript. 18S, 5.8S, and 28S rDNA genes form a transcription unit including ITS1 and ITS2. They are highly conservative and suitable for system analysis of the level of a higher level of groups of organisms. In addition, as fungal rDNA ITS sequences are conserved in evolution, the detection of this region helps to analyze the genetic relationship of different fungi. We analyzed the homology of ITS sequences among *H. sinensis* and other fungus species. It is found that *H. sinensis* has a very low homology with ordinary fungi but approximately 100% homology with *O. sinensis*. It indicates that this strain has region-specific and species-specific characteristics, and is the only correct anamorph of *O. sinensis*.

### Secondary metabolism analysis

Bioinformatics software such as DNAMAN and Primer 5.0, and online tools, such as KEGG PATHWAY Database (http://www.genome.jp/kegg/pathway.htmL), Blast (http://blast.ncbi.nlm.nih.gov/) and ORF Finder (http://www.bioinformatics.org/sms/orf_find.htmL) were used to analyze synthesis pathways of 7 main bioactive components (mannitol, cordycepin, purine nucleotides, pyrimidine nucleotides, unsaturated fatty acid, cordyceps polysaccharide and sphingolipid) in *H. sinensis*.

## Results

### Isolation and identification of *H. sinensis*

**Fig. 1:**
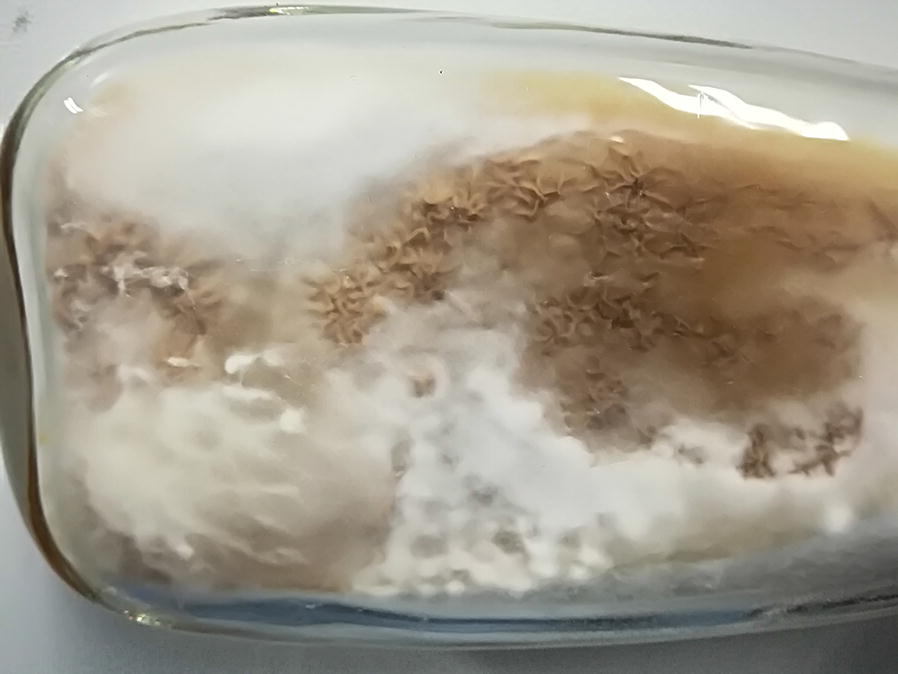
Colonial morphology of *H. sinensis*

When germinated after about 15 days, the cultured tissues were inoculated to liquid PDA medium by pure culture at 16 °C with 120 rmp. The culture on medium became pale yellow and a little bit thicker after 15 days. And the inoculated tissue surface was covered with white mycelia. After 30 days of culture, liquid mycelia were seeded into solid PDA medium, and the surface was covered with about 3 cm stromata after 20 days (Fig. 1). Finally, several species identification methods, such as molecular identification, biolog identification and morphological identification were carried out to identify whether the isolated strains were *H. sinensis*.

**Fig. 2:**
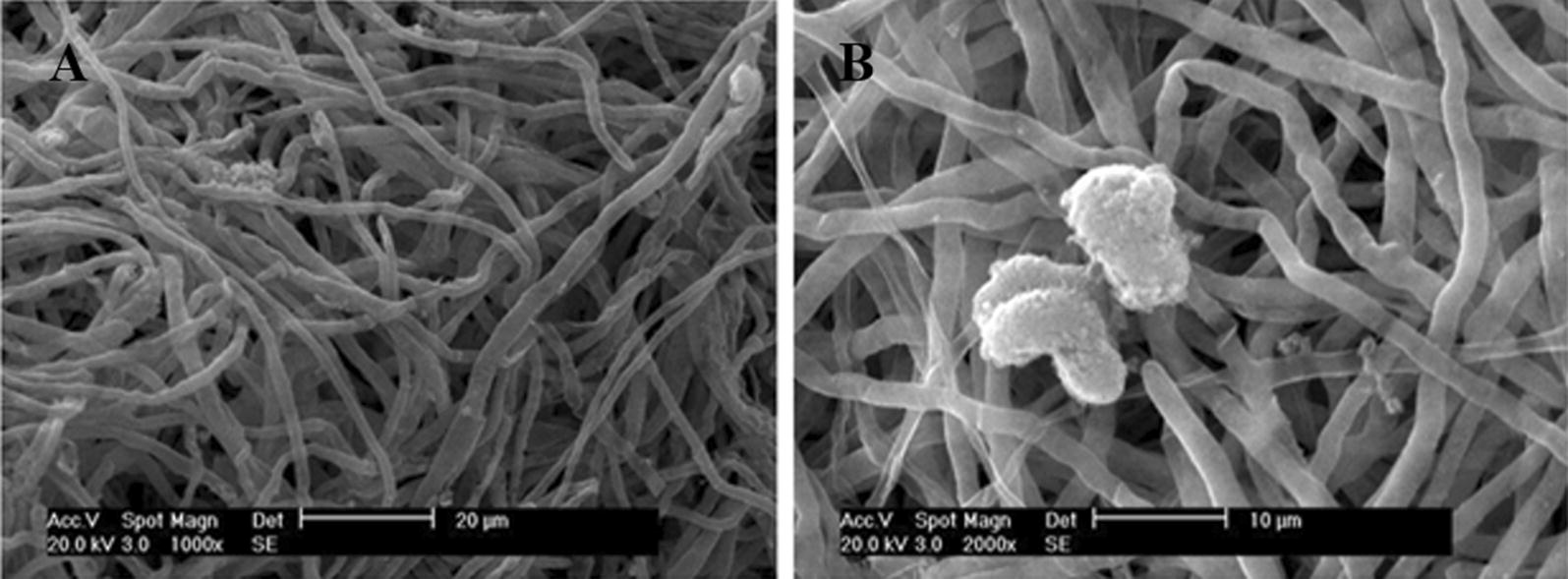
Electron micrograph of *H. sinensis.***a** ×1000; **b** is ×2000

**Fig. 3:**
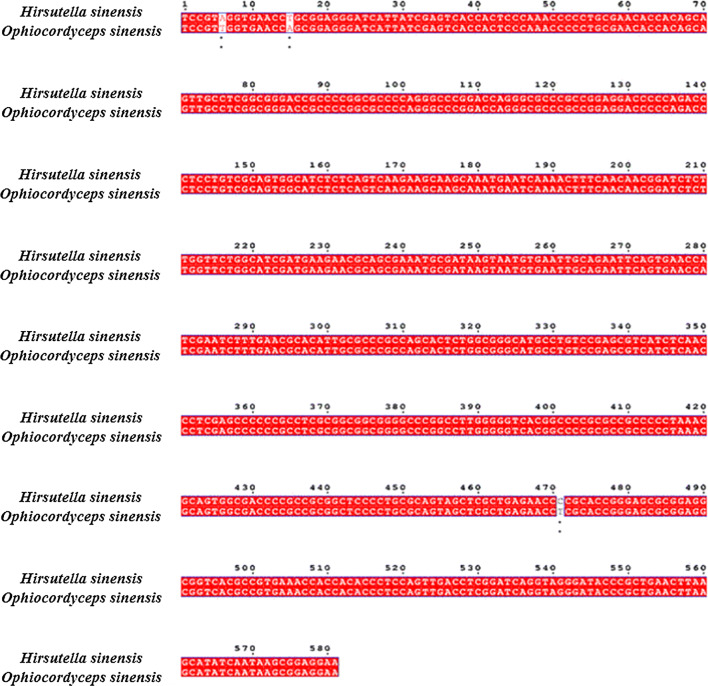
Sequence alignment of 18S rDNA between the isolated strain and *O. sinensis*

Morphological identification was firstly performed. Single colony formed on the plate was white, and the white mycelia were on the surface. Hyphae, as well as the formation of spores, were observed under electron microscope (Fig. 2). Subsequently, molecular identification was conducted. Genomic DNA from single colony was extracted under the protocol of Multisource Genomic DNA Miniprep kit (Axygen, USA) with minor modification. ITS1 and ITS4 primers were used to amplify ITS sequence, and the amplified ITS sequences were sequenced and aligned in NCBI. The obtained 18S rDNA sequence was subjected to homology analysis, and the results are shown in Fig. 3. The 18S rDNA sequence of the identified microorganism has the highest homology with *O. sinensis* (homology, 99%/539 bps, based on 18S rDNA), according to the molecular genetics identification principles, based on the 18S rDNA sequence homology of more than 95%, the identified fungal basically belongs to the control fungal.

**Fig. 4:**
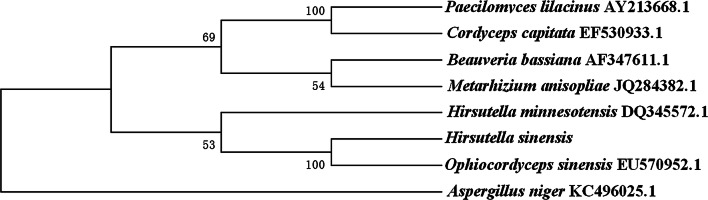
The phylogenetic tree of *H. sinensis*

In the last, the phylogenetic analysis was carried out (Fig. 4). It was shown that this strain had 100% homology with *O. sinensis*. Furthermore, it was analyzed by Biolog metabolic fingerprint. Results show that this strain can use 26 kinds of carbon sources, however, it can weekly use or not use other 69 kinds of carbon sources (Additional file 1: Table S1). After these procedures, it can be determined that the anamorph of *O. sinensis* were successfully isolated and was called *H. sinensis*.

### Genome sequencing and analysis

For the whole-genome shotgun sequencing of *H. sinensis* CCTCC M 2011278, we used Illumina Genome Analyser sequencing technology (Kensuke et al. 2011). After DNA was extracted using a standard phenol/chloroform method, 16 paired-end sequencing libraries were constructed with insert sizes of about 250 base pairs (bp), 350 bp, 500 bp, 5 kb, 6 kb, and 10 kb. A total of 28.97 Gb clean data was generated.

The genome was assembled using a SOAPdenovo, genome assembly software specifically designed for short-read sequences 1. We first assembled small fragments (< 500 bp) into 23,198 contigs with a N50 of 11,379 bp using sequence overlap information. These contigs were then assembled into 655 scaffolds (N50 = 3432). Finally, the scaffolds were assembled into unigenes. The short reads for each Index data were assembled into part of genome sequence using SOAPdenovo. Assembly statistics results were shown in Table 1.

**Table 1:** Assembly statistics results

	Scaffold	Contig
Total num (#)	655	23,198
Total length (bp)	102,604,129	96,613,874
N50 (bp)	343,253	11,379
N90 (bp)	79,506	2338
Max length (bp)	1,919,695	86,294
Min length (bp)	1962	71
Sequence GC (%)	45.83	45.83

This assembly strategy revealed a 102.72 Mb genome. The coding region of the genome constitutes 16.25% of the entire genome, which resulted in the gene density of 99 genes/Mb. The percent GC content of the coding region is significantly higher (60.19%) than the entire genome (45.83%) (Table 2). Gene annotation was based on the principle that the size of the minimum open reading frame (ORF) was 100 amino acids, which revealed 10,200 ORFs, with an average length of 1506 bp. Based on these ORFs we predicted a total number of 10,200 genes, which is slightly fewer than the proteins encoded by *M. acridum* genome, but more than the proteins encoded by the genomes of both *C. militaris* and *M. anisopliae* (Table 2). On average, each gene contains 2.85 exons and 1.85 introns, respectively. Blastp matches were identified with 9081 of the ORFs in at least one of the following databases: KEGG, COG, SwissProt, TrEMBL, NR and GO.

**Table 2:** Comparison of genome features among six insect pathogens

Features	*C. militaris*	*M. anisopliae*	*M. acridum*	*A. niger*	*P. chrysogenum*	*H. sinensis*
Size (Mb)	32.2	39.0	38.1	33.9	32.2	102.72
Coverage (fold)	147×	100×	107×	7.5×	2.3×	288.77×
G + C content	51.4	51.5	20.0	50.4	48.9	45.83
repeat rate	3.04	0.98	1.52	–	–	24.7
Protein-coding genes	9684	10,582	9849	14,165	13,653	10,200
Gene density (genes per Mb)	257	271	259	420	402	99
Exons per gene	3.0	2.8	2.7	2.57	3	2.85
Secreted proteins	16.2	17.6	15.1	–	–	–
tRNA	136	141	122	269	145	122
Pseudogenes	102	363	440	–	592	–
NCBI accession	AEVU00000000	ADNJ00000000	ADNI00000000	ACJE00000000	NS_000201	LWBQ00000000

### Genome general features

The genome is larger than the genomes of the pathogen of lepidopteran insect pupaethe *Cordyceps militaris* (CCM), the broad host range *Metarhizium anisopliae* (MAA) and the locust-specific pathogen *Metarhizium acridum* (MAC) that sequenced previously (Table 2). From mapping > 5000 expressed sequence tags, the *H. sinensis* genome was estimated to be > 99% complete. Consequently, the genome was predicted to encode 10,200 protein genes.

### Non-coding RNA prediction

Non-coding RNA (ncRNA) widely exists in bacteria, archaea and eukaryote (Eddy 2001; Kensuke et al. 2011). They participate in many biological functions but do not encode proteins. NcRNA contains sRNA, rRNA, tRNA, snRNA and microRNA etc. (Knoll et al. 2015; Kouji et al. 2009; Palazzo and Lee 2015). The results (Additional file 1: Table S2) indicated that *H. sinensis* genome contains sRNA, rRNA, tRNA and snRNA, which has 122, 35, 5 and 33 copy numbers, respectively. The average length of ncRNA ranges from 88 to 595 bp.

### Repetitive sequence analysis

RepeatMasker (using Repbase database) and RepeatProteinMasker (using the RepeatMasker library that comes with transposon protein) are two popular softwares to predict the transposon. The transposon sequences via aligning assembly result. Tandem repeats were predicted by TRF (Tandem Repeat Finder) software (Kapitonov and Jerzy 2008; Tempel and Sébastien 2012). The results were shown in Additional file 1: Table S3. Aligning assembly result with RepeatProteinMasker software shows a 18,546,115 bp repeat which account for 18.08% of Genome, while the ratio of repeats results from RepeatMasker software and TRF was 7.4% and 6.1%, respectively. Finally, the redundant sequences found in the above three databases were de-redundant to obtain 25,410,472 bp repeats, accounting for 24.7% of the total genome.

### Gene prediction

425 genes are predicted by *CEGMA* software in the genome. Generally, there are 405 genes predicted by *CEGMA* software in the fungus. It shows the gene regions in the genome that have been assembled. We predicted genes by *Homology* with several Homologous species, including *Chaetomium globosum*, *Fusarium oxysporum*, *Neurospora crass*, *Saccharomyces cerevisiae* and *Penicillium chrysogenum Wisconsin*. From the prediction results, the number of genes in *Saccharomyces cerevisiae* is fewer, which means that it has longer genetic distance between *S. cerevisiae* and the target species. So we choose the result of the other four species as the result of *Homology*. We merged the de novo and *Homology* predictions and the assembly sequence together with glean software to obtain the primary prediction result. Finally, the gene numbers of 10,200 (29,077 Exons and 18,877 Introne) was obtained. The results were shown in Tables 3 and 4. The average gene length was 1506 bp with a 60.19% GC content, and the ratio for gene in genome is 16.25%. For intergenic region, they account for 83.47% of genome with the length of 85,925,228 bp.

**Table 3:** Gene prediction stat

Gene number	10,200
Gene length (bp)	16,678,901
GC content in gene region (%)	60.19
Gene/genome (%)	16.25
Gene average length (bp)	1506
Intergenic region length (bp)	85,925,228
GC content in intergenic region (%)	42.89
Intergenic length/genome (%)	83.74

**Table 4:** Gene prediction stat

	Gene stat	Exons stat	CDS stat	Introne stat
Total length (bp)	16,678,901	12,065,133	12,065,133	2,692,792
Total number	10,200	29,077	10,200	18,877
Average length (bp)	1506.10	414.94	1182.86	142.65

### Gene function annotation

**Fig. 5:**
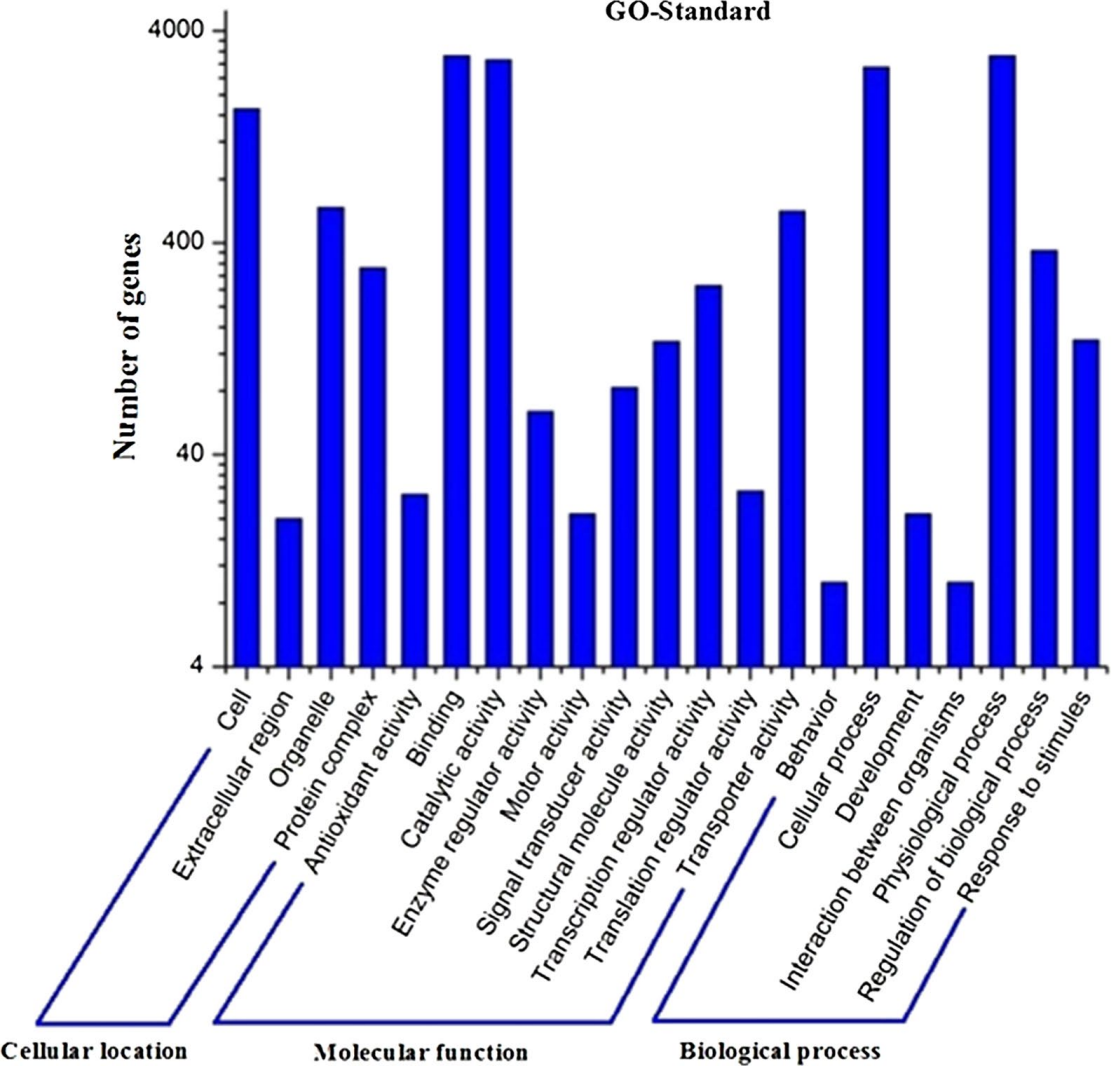
KEGG pathway classification

**Fig. 6:**
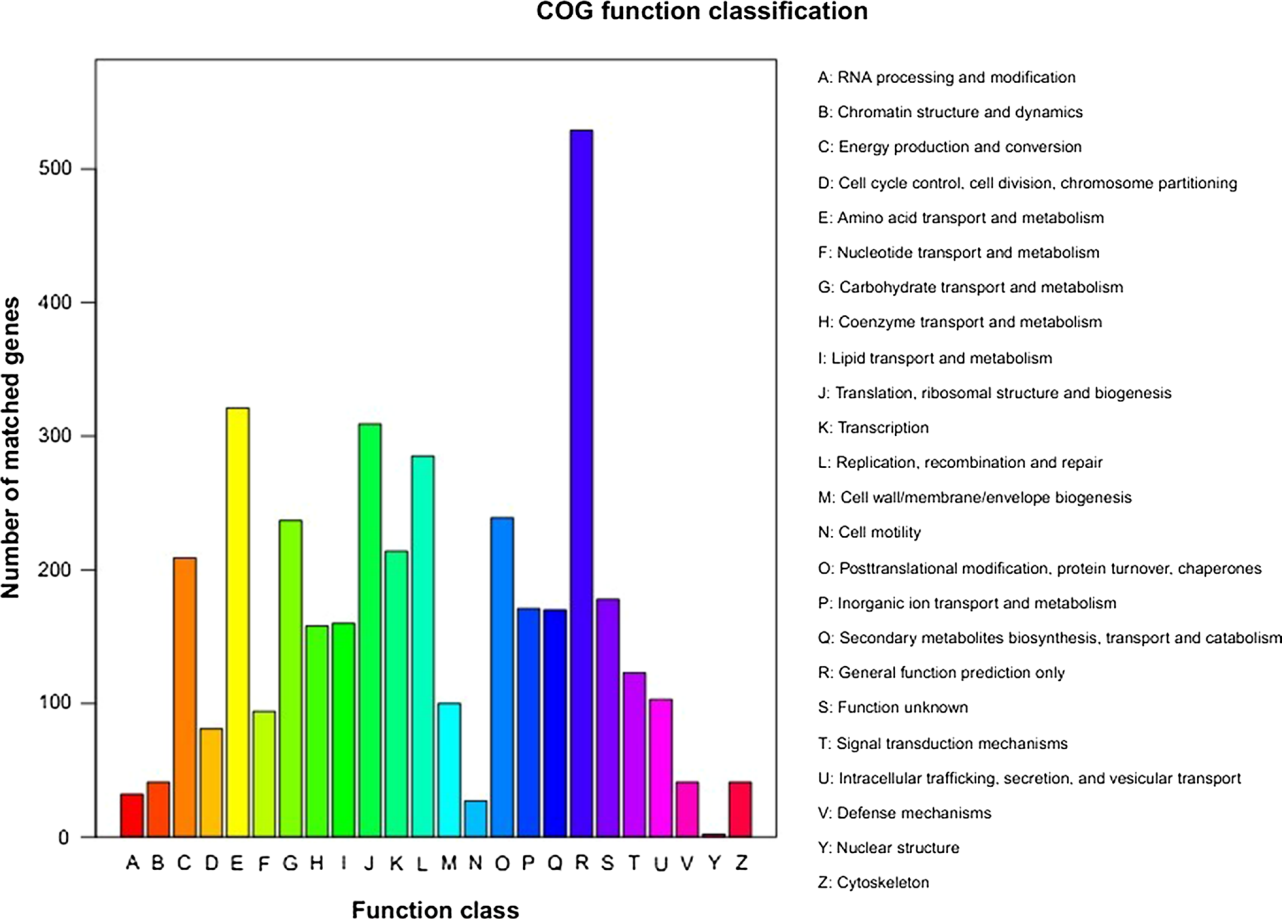
COG function classification histogram

**Fig. 7:**
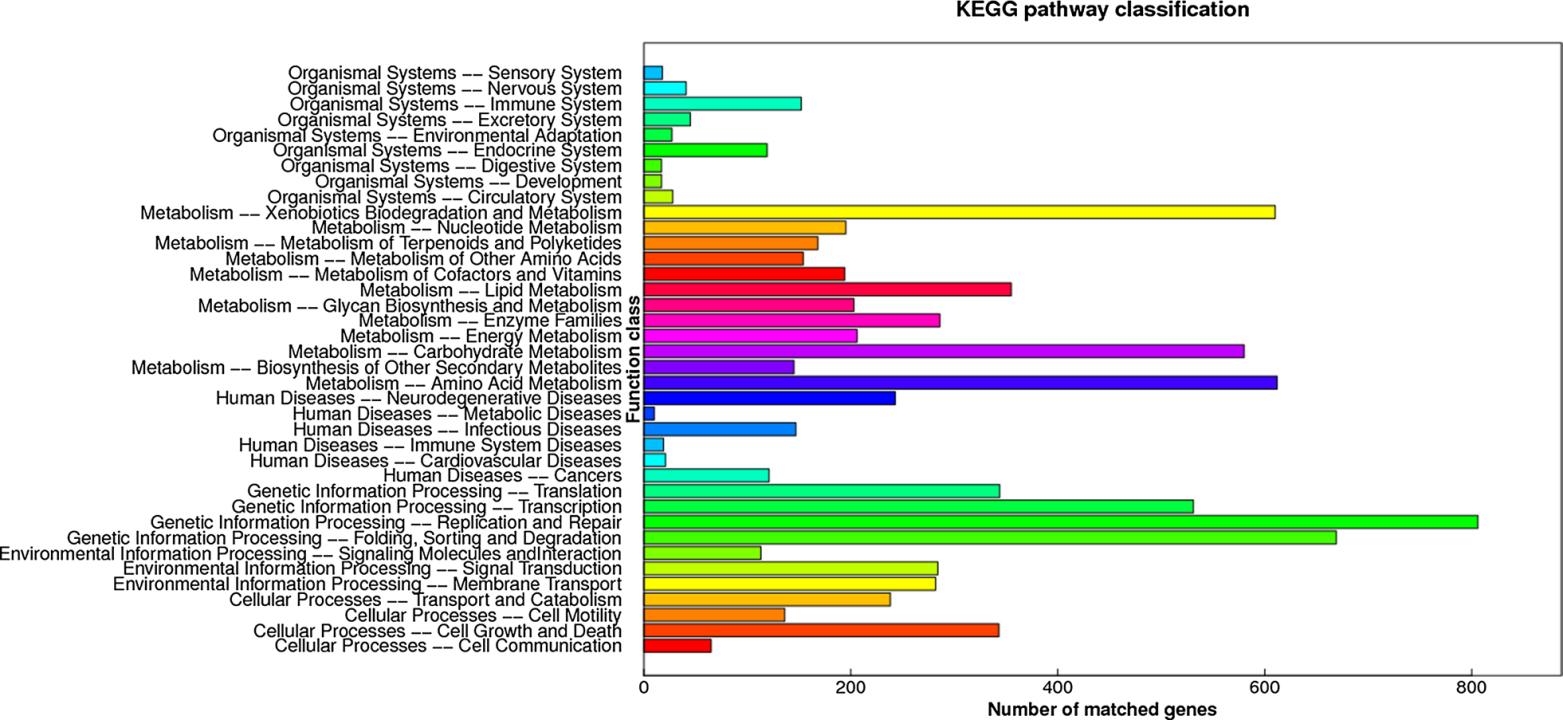
GO classification histogram

In GO assignments, genes were categorized into 21 functional groups. In terms of the biological process, genes involved in ‘cellular process’, ‘physiological process’, ‘regulation of bilogical process’ accounted for the majority. For molecular function, genes involved in ‘binding’, ‘catalytic activity’ accounted for the majority (Fig. 5). The annotated sequences were taken for the genes involved in COG classifications. In 26 COG categories, the cluster for ‘general function prediction’ represents the largest group followed by ‘amino acid transport and metabolism’ and ‘lipid transport and metabolism’ (Fig. 6). To identify the biological pathways in *H. sinensis*, the annotated genes were mapped to the reference canonical pathways in KEGG, the most representative pathways were ‘replication and repair’, ‘sorting and degradation’, ‘xenobiotics biodegradation and metabolism’ and ‘amino acid metabolism’ (Fig. 7). These annotations provide a valuable resource for investigating specific processes, functions and pathways in *H. sinensis*.

**Fig. 8:**
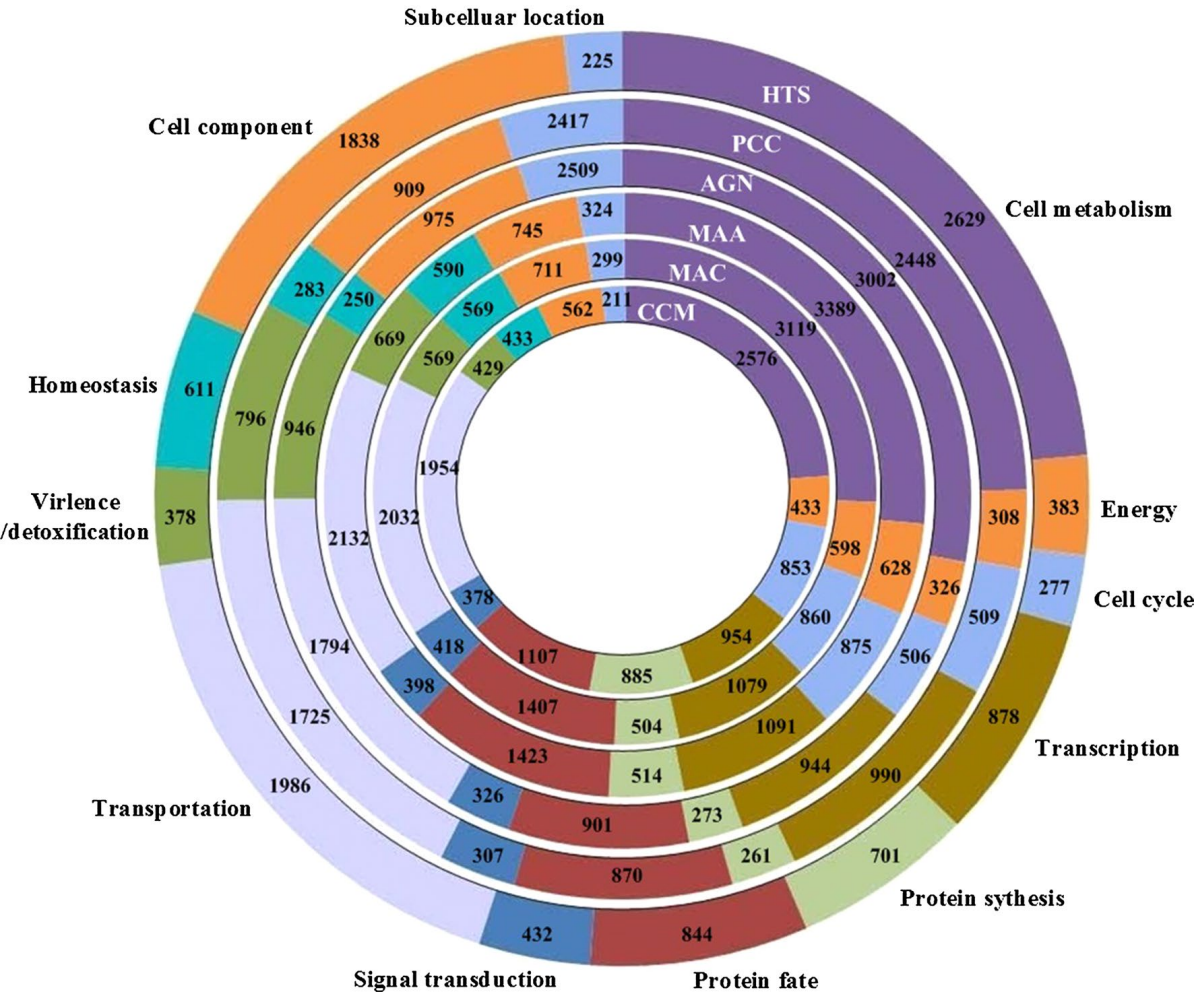
Functional classification and comparison of *C. militaris* (CCM), *M. anisopliae* (MAA), *M. acridum* (MAC), *A. niger* (AGN)*, P. Chrysogenum* (PCC) *and H. sinensis* (HTS) proteins, showed that *H. sinensis* has different genes in each category. Each circle represents the relative fraction of genes represented in each of the categories for each genome

We also carried out functional classification and comparison of *C. militaris* (CCM), *M. anisopliae* (MAA), *M. acridum* (MAC), *A. Niger* (AGN), *P. Chrysogenum* (PCC) with *H. sinensis* (HTS) genes. As the results were shown in Fig. 8, the gene amount of cell component, subcellular location, virulence, protein synthesis and cell cycle for different fungus varied apparently, which indicated the discrepancy of growing environment and life cycle of these fungus. Gene number for transportation, signal transduction, protein fate, transcription, energy and cell metabolism was relatively constant. For “Cell component”, *H. sinensis* had 1838 genes that was far more than that in *C. militaris*, *M. anisopliae*, *M. acridum*, *A. Niger*, *P. Chrysogenu*, which may be due to its more complex cell composition under the conditions of low temperature, low pressure and high ultraviolet.

### Protein family analysis

Gene family expansions for glucanases, proteases, phospholipases, chitinases and laccases in *H. sinensis* were identified after comparison with other phytopathogenic fungi. Proteases were the largest family expansions. The *H. sinensis* genome contains 37 proteases but most of them belong to families of serine proteases or cysteine protease. However, different families of proteases are also expanded in *Metarhizium* spp, *C. Militaris*, *A. niger* and *P. chrysogenum*, respectively. The second largest family expansions were chitinases, which contains 31 chitinases. As for other families, glucanases contained 13, phospholipases contains 15, and laccases contained 7, respectively.

### Secondary metabolism analysis and verification

Then functional genes and proteins were confirmed which participate in synthesis process in *H. sinensis* using gene cloning and protein expression methods. Since they provided a reference to verify the correctness of metabolic pathways, it will help to provide new information and method for further regulation, control and optimization the fermentation process to improve *H.sinensis* quality. In addition, it will provide us a novel way to enhance the genetic stability and improve functional active components of *H.sinensis*.

Mannitol and its deviates are important active compounds in *H. sinensis*. Mannitol is a good diuretic in medicine with the function of preventing acute renal failure. It is also a preferred drug for treating cerebral edema and can reduce intracranial pressure by dehydration. We predicted the pathway from glucose to mannitol according to the glycolytic pathway (map00010) and fructose-mannose pathway (map00051) annotated by KEGG metabolic pathway. However, mannitol-1-phosphatase that converts mannitol-1-P to mannitol was not found. This result indicates that mannitol-1-P is the final product in mannitol metabolic pathway.

**Fig. 9:**
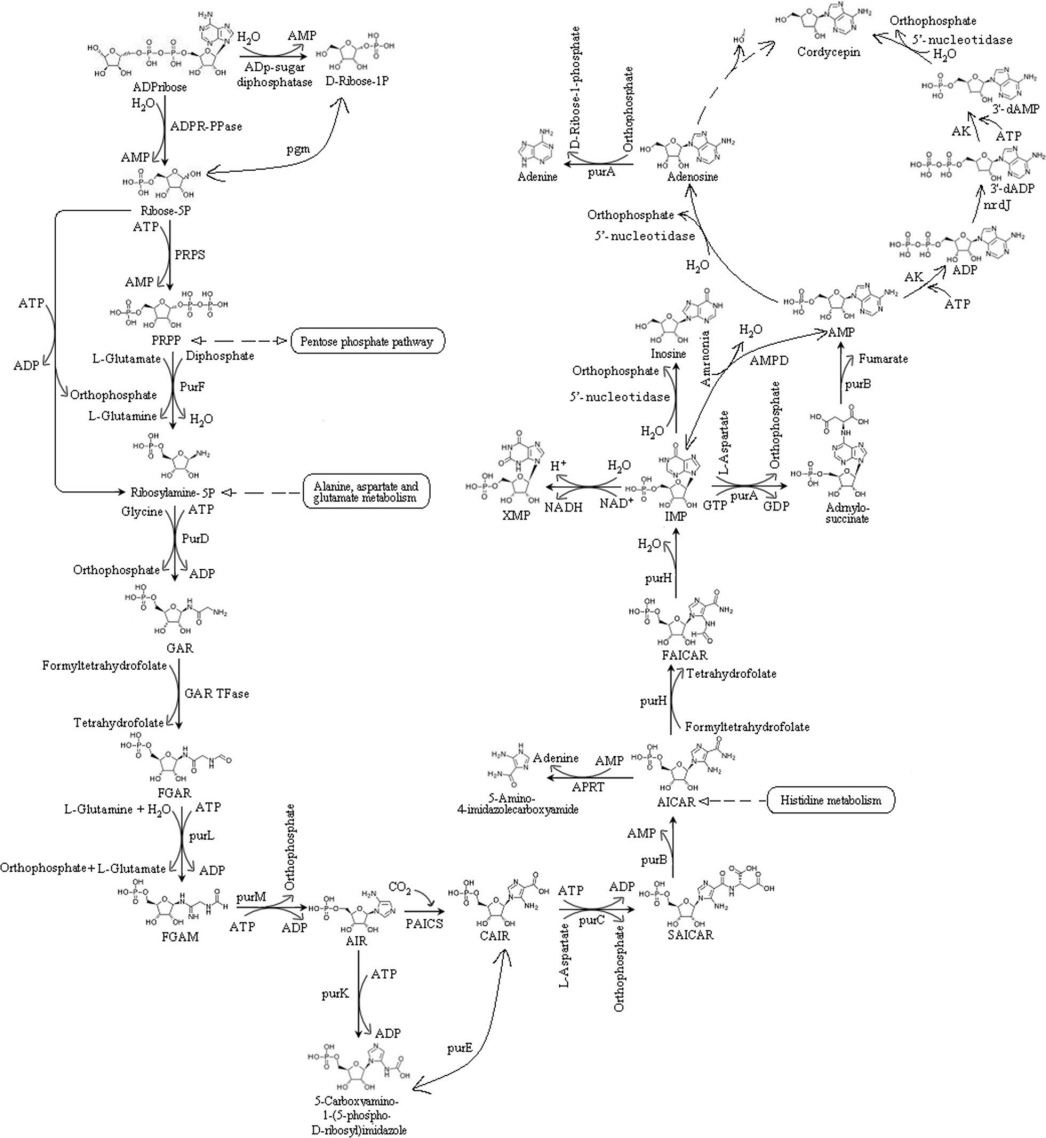
The cordycepin anabolism pathway in *H. sinensis*

Cordycepin is one of the most important active components in *H. sinensis.* Cordycepin not only has anti-tumor, anti-leukemia, immune regulation and free radical clearance functions, but also has antibacterial, anti-inflammatory, antiviral, decreasing blood sugar and anti-aging effects. The content of cordycepin in *H. sinensis* was examined by LC–MS analysis (Additional file 1: Figure S1). In the KEGG metabolic pathways, the synthesis of adenosine (map00230) has been confirmed. However, there was still no literature to report for the synthesis of cordycepin from adenosine. We speculated a biological metabolic pathway from adenosine to cordycepin. Cordycepin is finally generated by the replacement of phosphate groups and hydroxyl groups by 3′-deoxyadenosine monophosphate under the action of 5′-nucloetidase. In summary, cordycepin synthesis pathway can be described as follows: First, ADP-ribose pyrophosphatase catalyzes the conversion from ATP ribose to 5-P-ribose; then, ribose-phosphate pyrophosphokinase catalyzes the reaction from 5-P-ribose to phosphoribosyl pyrophosphate; next, Ribosylamine-5P was generated from phosphoribosyl pyrophosphate by the catalysis of amidophosphoribosyl transferase. Then, phosphoribosylamine-glycine ligase catalyzes the conversion from 5-phosphoribosyl amine to glycinamide ribonucleotide, and phosphoribosyl glycinamide formyltransferase catalyzes the generation of *N*-fomylglycinamide ribotide from glycinamide ribonucleotide. In the remaining steps, *N*-fomylglycinamide ribotide successively goes through a 10 step reaction to generate adenosine. In the last step, cordycepin is produced from 3′-deoxyadenosine monophosphate by 5′-nucloetidase. Subsequently, corresponding proteins were expressed in *E. coli* BL21 and examined by protein electrophoresis (Additional file 1: Figure S2). The proposed biosynthesis pathway of cordycepin is shown in Fig. 9. Moreover, cordycepin in *H. sinensis* was detected by LC–MS, as shown in Additional file 1: Figure S1. Cordycepin standards were firstly detected by LC–MS, the retention time and m/z were shown in Additional file 1: Figure S1A, B. Subsequently, cordycepin in *H. sinensis* was extracted and analyzed. According to the comparison of retention time and m/z between cordycepin standards and sample, we confirmed the structure of cordycepin in *H. sinensis.*

Purine has antiviral and anti-tumor pharmacological effects. According to KEGG metabolic pathway annotation of *H. sinensis* transcriptome, the purine metabolic pathway (map00230) is from adenosine to adenine, adenosine monophosphate, inosine, guanosine monophosphate, guanosine, guanine, etc. Relevant enzymes include: (1) purine nucleosidase, (2) adenosine kinase, (3) adenine phosphoribosyl transferase, (4) AMP deaminase, (5) IMP dehydrogenase, (6) GMP synthase, (7) guanine deaminase, (8) xanthine dehydrogenase, and (9) 5′-nucleotidase. Pyrimidine also has antiviral and anti-tumor pharmacological effects. According to KEGG metabolic pathway annotation for pyrimidine metabolism (map00240), we proposed the synthesis pathway from l-glutamine to uridylic acid, cytidylic acid, thymidylic acid etc. Also, the genes involved in pyrimidine pathways were identified and cloned (Additional file 1: Figure S3). Subsequently, corresponding proteins were expressed in *E. coli* BL21 and examined by protein electrophoresis (Additional file 1: Figure S4). The biosynthesis pathway of pyrimidine started from l-glutamine. Through transformation of carbamoyl-P to *N*-carbamoyl-l-aspartate, dihydroorotate, orotate, orotidine-5P, uridylic acid was obtained. Cytidylic acid and thymidylic acid were taken by the catalysis reaction from uridylic acid.

**Fig. 10:**
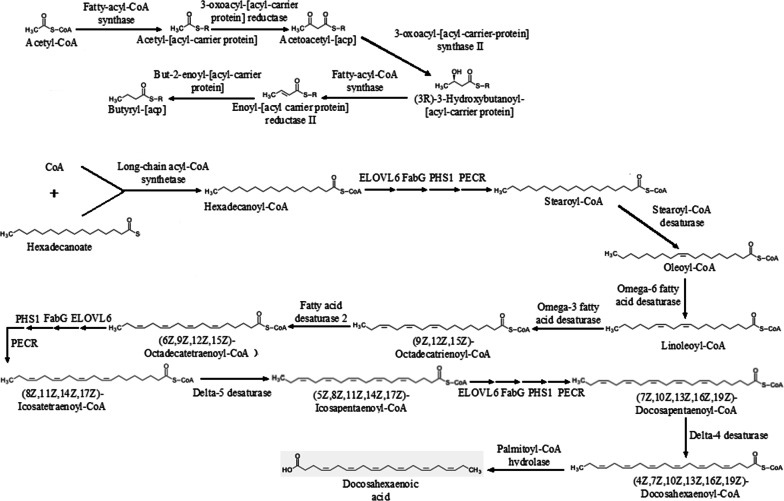
The fatty acid anabolism pathway in *H. sinensis*

Unsaturated fatty acid is the precursor to synthesize prostaglandins and thromboxane in humans. It can reduce cholesterol in blood by esterifying cholesterol, and reduce blood viscosity to improve blood circulation. The synthesis pathway from acetyl-CoA, CoA and hexadecanoate to docosahexaenoic acid and butyryl-acp was built, as shown in Fig. 10, based on the biosynthesis pathways of fatty acid (map00061) and unsaturated fatty acid (map01040) in KEGG database. GC–MS analysis revealed three kinds of unsaturated fatty acid in *H. sinensis*: hexadecanoic acid, oleic acid, and linoleic acid (Additional file 1: Figure S5). The verification of the predicted unsaturated fatty acid metabolic pathways was further studied. Genes involved in these pathways were identified in the genome of *H. sinensis*. Genes of enzymes in metabolic pathways were successfully cloned for unsaturated fatty (Additional file 1: Figure S6) metabolic pathways, respectively. Corresponding proteins were expressed in *E. coli* BL21 and examined by protein electrophoresis (Additional file 1: Figure S7).

**Fig. 11:**
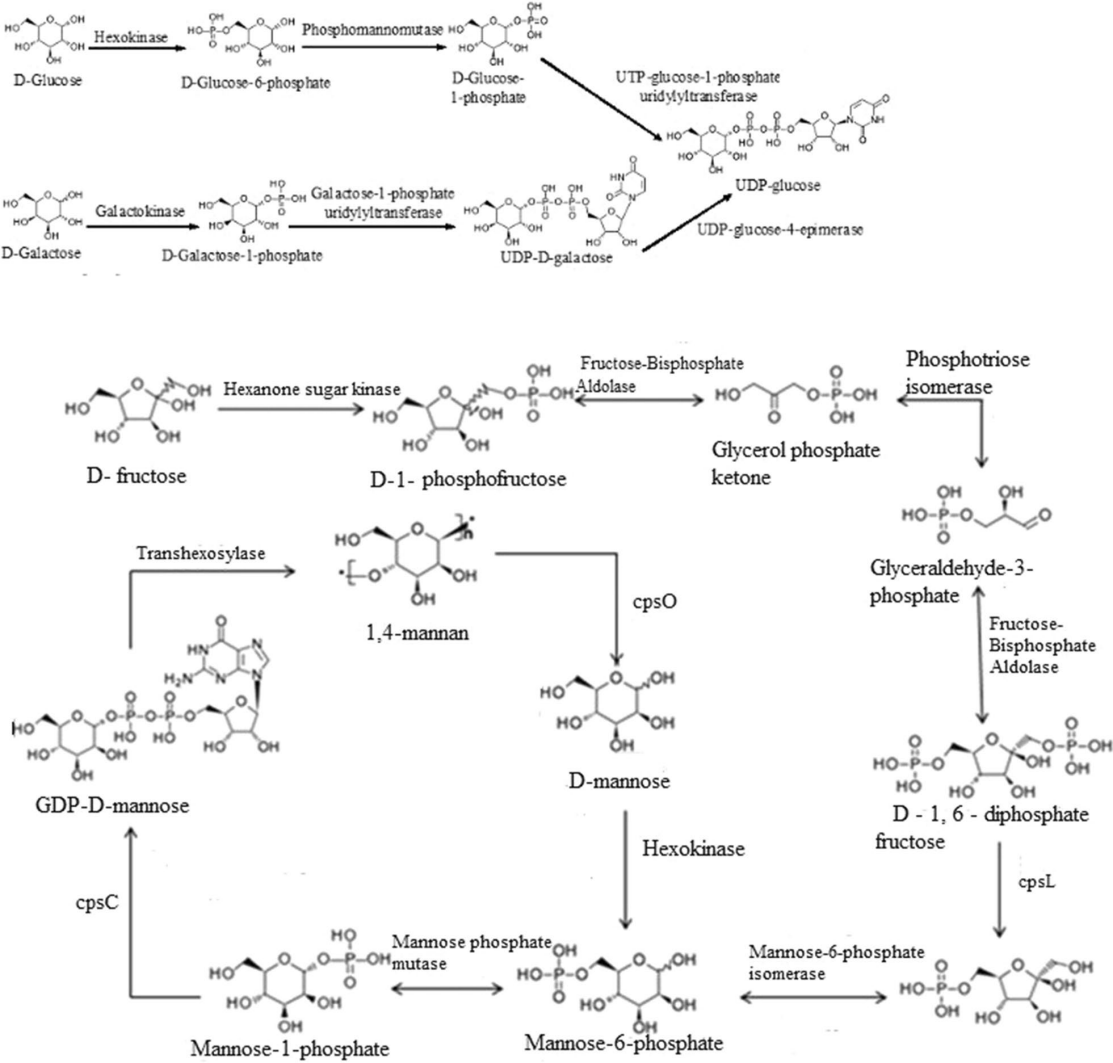
The cordyceps polysaccharide anabolism pathway in *H. sinensis*

Cordyceps polysaccharide is a kind of non-specific immune accelerators which can improve immune function. In addition, it also has pharmacological effects of anti-tumor, anti-aging, decreasing blood sugar, anti-radiation and protecting kidneys. According to the KEGG fructose and mannose metabolism pathway (map00051), galactose metabolism pathway (map00052) and *N*-glycan biosynthesis pathway (map00510), we supposed the synthesis pathway from d-glucose, d-galactose and dolichol phosphate to UDP-glucose and (GlacNAc)2(Man)5(Asn)1, as shown in Fig. 11. Further analysis of cordyceps polysaccharide revealed that the polysaccharide is composed of d-mannose, d-galactose and d-Glucose (Additional file 1: Figures S8–S12). The genes involved in cordyceps polysaccharide pathways were identified in the genome of *H. sinensis*. Genes of enzymes in metabolic pathways were successfully cloned for cordyceps polysaccharide (Additional file 1: Figure S13) metabolic pathways. Corresponding proteins were expressed in *E. coli* BL21 and examined by protein electrophoresis (Additional file 1: Figure S14).

Sphingolipid is related to the specificity of blood group, histogenic immunity and cell recognition. It is also involved in many other important signal transduction processes such as regulation of cell growth, differentiation, senescence and programmed cell death etc. According to the KEGG sphingolipid metabolism pathway (map00600), we obtained the synthesis pathway from palmitoyl CoA and serine to sphingosine and phytoceramide.

### The enzymes involved in the infection mechanism

Generally, when infecting the host, *H. sinensis* produces some enzymes including glucanase, protease, phospholipase, chitinase and laccase to degrade the cell wall, cell membrane and intracellular part of host (Table 5). It is well known that the protease can be subdivided into serine proteases, arginine protease, cysteine protease, phospholipase and cuticle-degrading protease (Table 5). These enzymes can degrade the cell wall, which contains chitin, proteins, lipids and other ingredients to facilitate the invasion.

**Table 5:** The enzymes involved in the infection mechanism

Enzyme	Numbers in transcription	Numbers in genome	Classification
Glucanase	72	42	Endoglucanase
			Exoglucanase
Protease	31	26	Serine protease
			Arginase
			Cysteine protease
			Cuticle-degrading protease
Chitinase	52	23	Endochitinase
			Exochitinase
Phospholipase	74	65	Phospholipase A1
			Phospholipase A2
			Phospholipase B
			Phospholipase C
			Phospholipase D
Laccase	10	7	I type copper ions laccase
			II type copper ions laccase
			III type copper ions laccase

In order to verify the enzymes involved in the infection mechanism, we screened the annotation results of sequenced genome of *H. sinensis*. Subsequently, 31 chitinase genes and 37 protease genes were found, bioinformatic analysis was carried out. Signal peptides of the enzymes were predicted and removed. Finally, 1 serine protease, 1 arginine protease, 3 chitinases, 1 phospholipase and 1 laccase were successfully cloned and expressed in *E. coli* BL21 (Additional file 1: Figure S15), and the corresponding enzyme activities were also examined.

## Discussion

Most of the current studies focused on the medical applications and mechanisms of *O. sinensis*, little is known for the genetic information of this organism, which is an essential tool for detailed understanding of the biology of this organism. The fungal genome and physiological characteristics are more complex than those of bacteria, so fungal genomics research has lagged behind bacteria. With the widespread application of next-generation sequencing technology in the field of biology, it has become easier to carry out the sequencing of larger genomes, and the genome research of fungi has become widespread.

In recent years, the genome research of entomogenous and medicinal fungi has become more and more extensive. Gao et al. (2011) performed a genome sequencing comparison of the model entomogenous fungi *Metarhizium anisopliae* and *M. acridum*. He found that the genome size of *M. anisopliae* was 39.0 Mb, the sequencing depth was 100×, and the GC content was 51.5%, there are 10,582 protein-coding genes in total, and 3389 genes are included in the category of cell metabolism, accounting for 32%; the genome size of *M. acridum* is 38.1 Mb, the sequencing depth is 107×, the GC content is 50.0%, there are 9849 protein-coding genes, and 3119 genes are included in the functional metabolism of cells, accounting for 31.7%. Zheng et al. (2012) sequenced the genome of *Cordyceps militaris* Cm01. This study sequenced the genome of *Cordyceps* for the first time. The results showed that the genome size of *Cordyceps militaris* was 32.2 Mb, the sequencing depth was 147×, and the GC content was 51.4%. There were 9684 protein-coding genes, and 2576 genes were included in the cell metabolism category, accounting for 26.6%. Hu et al. (2013) sequenced the genome of *O. sinensis* Co18 isolated from the fruit body of *Cordyceps sinensis*, the results show that its genome size is about 120 Mb, and its sequencing depth is 241×, which is significantly higher than the above three entomogenous fungi. The GC content is 46.1%. There are 6972 protein-coding genes in total. The functional genes involved in the infection mechanism, secondary metabolism, and cold tolerance mechanism were preliminary discussed and studied. In this study, we sequenced the genome of *H. sinensis* and 10,200 protein-encoding genes were predicted based on a 102.72 Mb genome sequence. The results showed that GC contents is normal and there are 10,200 protein-coding genes, but the gene repeat rate is significantly higher and the Gene density is significantly lower, which also explained that its whole genome is larger than the other five entomogenous fungi.

The main biological and pharmacological active ingredients in *H. sinensis* are mannitol, cordycepin, purine nucleotides, pyrimidine nucleotides, unsaturated fatty acid, cordyceps polysaccharide and sphingolipid. Cordycepin is a functional active ingredient in *H. sinensis*, it is an important indicator for the quality control of *H. sinensis* fermented products. The cordycepin biosynthetic pathway has important guiding significance for improving cordycepin production. Lennon and Suhadolnik (1976) used [U-^14^C] adenosine and [3-^3^H] ribose to study the biosynthetic pathway of cordycepin, and the results showed that 2′-deoxyadenosine may be a precursor substance of cordycepin biosynthesis. Zheng et al. (2012) sequenced the genome of *Cordyceps militaris*, inferred the biosynthesis process of cordycepin by constructing a metabolic pathway of purine and adenosine, and found that 5′-nucleotidase is a key enzyme in cordycepin biosynthesis. The above studies provide important references for the construction of *H. sinensis* biosynthesis pathway. In this study, we annotated the KEGG pathway after sequencing the *H. sinensis* genome and assembling the short reads, analyzed the biosynthetic pathways of *H. sinensis* and studied the sequences of related enzymes. By searching and screening the metabolic pathways of the target metabolic end products from the KEGG Pathway Database, the metabolic pathway map number was obtained; then the KEGG Pathway annotation information database was used to find the annotation pathway corresponding to the pathway number; by comparing and analyzing the online KEGG Pathway Database and the annotated information of *H. sinensis* genome KEGG Pathway, the biosynthetic pathways of secondary metabolites and the sequences of related enzymes were studied. A detailed secondary metabolism analysis of the main ingredients was developed, and the biosynthesis pathways of seven ingredients (mannitol, cordycepin, purine nucleotides, pyrimidine nucleotides, unsaturated fatty acid, cordyceps polysaccharide and sphingolipid) were established according to the sequencing results of genome.

As the asexual type of *O. sinensis*, in the nature, *H. sinensis* mainly infects host larvae in the soil through the skin of the head by the effect of enzymes and mechanical force (Zacharuk 1971). The entire process includes host recognition, mechanical damage, nutrient competition, metabolism disturbance, secretion of toxins and damage of host tissue structure. These multi-factor interactions ultimately lead to host death and form the matured *O. sinensis* (Taylor and Gurr 2014). Theoretically, the invasion can directly cause insect infection without depending on feeding activities of host. In this stage, hydrophobic spores passively attach to the hydrophobic insect body wall and the adhesion interaction is weak. The proteases are secreted from outside of spores, which enables the conidia to firmly attach to the body wall of the host (Holder and Keyhani 2005). During the infection, the appressorium is formed in the tip of germ tube, which is oval cells adhering to the skin and 2–3 times larger than conidium, it can secret enzymes including chitinase and protease to degrade the mucus and epidermis, which makes the appressorium lose waxy layer. In the process of insect body wall invasion, *H. sinensis* secretes many kinds of exocellular hydrolytic enzymes such as protease (Samuels and Paterson 1995), chitinase (Kim et al. 2019; St et al. 1996) and lipase etc. which all play important roles (Charnley and Leger 1991). Degradation of proteases is not only helpful to the mycelium penetration, but also provides nutrients for mycelial growth (Charnley and Leger 1991). By screening the genome annotation of *H. sinensis*, we predicted and found 118 proteases, 23 chitinases and 107 lipases. After penetrating into the hemocoel, *H. sinensis* first overcomes the immune protection response of host, and then effectively obtains nutrients from host body for growth and reproduction. To avoid host cellular immunity, *H. sinensis* in host hemocoel forms short rod-like hyphalbody or blastospores. Previous studies indicated that this kind of cells with few or no cell wall can avoid the host immune response caused by polysaccharides (Pendland et al. 1993). *H. sinensis* can overcome the host’s immune bactericidal effect not only because of its repackaging cell wall structure, but also because of its capability of secreting toxic secondary metabolites (also known as toxins to host) (Gillespie et al. 2000). Molecular weight toxins consist of enzyme-toxins and non-enzyme-toxins. Enzyme-toxins include trypsin-like protease, chymoelastase (Urtz and Rice 2000) and serine proteinase (Kim et al. 1999), while non-enzyme-toxins include glycoprotein (Mollier et al. 1994). In this study, we found that chitinase and proteases, especially serine proteases, showed significant gene family expansion, especially those of *H. sinensis* grown under low temperature conditions. The enzymes contained in *H. sinensis* are of special value. Our research provides a theoretical basis for the further development of valuable entomogenous fungal characteristic enzyme systems.

## Supplementary information

**Additional file 1**. Additional figures and tables.

## Data Availability

The annotated genome has been deposited at the NCBI database with the accession number LWBQ00000000.
