# Supplementary material for: Genome sequencing and analysis of fungus Hirsutella sinensis isolated from Ophiocordyceps sinensis
Source: AMB Express. 2020 Jun 3;10:105. doi: 10.1186/s13568-020-01039-x (PMC7270455; doi:10.1186/s13568-020-01039-x)
Supplement: Supplementary file 1 — Additional file 1. Additional figures and tables. [file 13568_2020_1039_MOESM1_ESM.docx]

**Additional file**

**Title: Genome sequencing and analysis of fungus *Hirsutella sinensis* isolated from *Ophiocordyceps sinensis***

Li-Qun Jin^1^, Zhe-Wen Xu^1^, Bo Zhang^1^, Ming Yi^1^, Chun-Yue Weng^1^, Shan Lin^1^, Hui Wu^2, 3^, Xiang-Tian Qin^2, 3^, Feng Xu^2, 3^, Yi Teng^2, 3^, Shui-Jin Yuan^2, 3^, Zhi-Qiang Liu^1^, Yu-Guo Zheng^1^

^1^Key Laboratory of Bioorganic Synthesis of Zhejiang Province, College of Biotechnology and Bioengineering, Zhejiang University of Technology, Hangzhou 310014, China

^2^HuaDong Medicine (Hangzhou) Bailing Biological Technology Co.,Ltd, Hangzhou 311220, China.

^3^East China Pharmaceutical Group Limited Co.,Ltd, Hangzhou 311000, China.

Email addresses:

Li-Qun Jin: jlq@zjut.edu.cn, Bo Zhang: [zhangbo0305@zjut.edu.cn](mailto:zhangbo0305@zjut.edu.cn), Zhe-Wen Xu: 570398426@qq.com, Ming Yi: 15958110983@163.com, Chun-Yue Weng: [cweng@zjut.edu.cn](mailto:cweng@zjut.edu.cn),Shan Lin: [biotechlin@foxmail.com](mailto:biotechlin@foxmail.com), Xiang-Tian Qin: [qinxt@eastchinapharm.com](mailto:qinxt@eastchinapharm.com), Hui Wu: [wuhui@eastprotech.com](mailto:wuhui@eastprotech.com), Feng Xu: [xufeng@hdpharm.com](mailto:xufeng@hdpharm.com), Yi Teng: [tengyi198777@126.com](mailto:tengyi198777@126.com), Shui-Jin Yuan: [shuijinyuan@eastchinapharm.com](mailto:shuijinyuan@eastchinapharm.com), Yu-Guo Zheng: zhengyg@zjut.edu.cn

**Author for correspondence**: Zhi-Qiang Liu,

Key Laboratory of Bioorganic Synthesis of Zhejiang Province, College of Biotechnology and Bioengineering, Zhejiang University of Technology, Hangzhou 310014, China

microliu@zjut.edu.cn.

**Additional figures**

**Figure S1: Agarose gel electrophoresis of resulting PCR fragment of the pyrimidine nucleosides anabolic functional genes from *H. Sinensis***

**
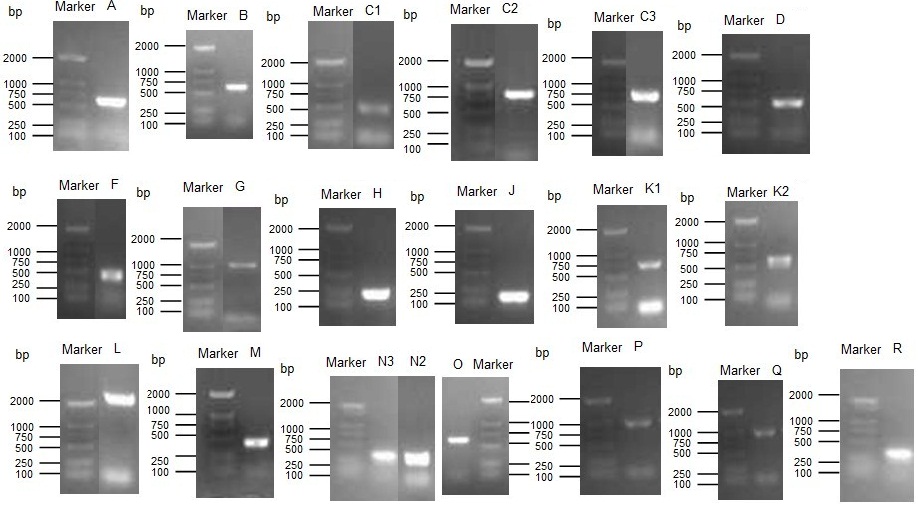
**

**Figure S2: Agarose gel electrophoresis of resulting PCR fragment of the unsaturated fatty acid anabolic functional genes from *H. Sinensis***

**
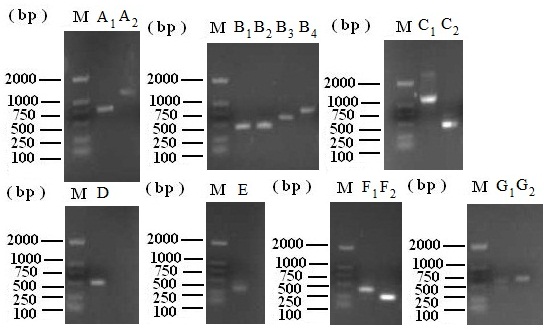
**

**Figure S3: Agarose gel electrophoresis of resulting PCR fragment of the cordyceps polysaccharide anabolic functional genes from *H. Sinensis***

**
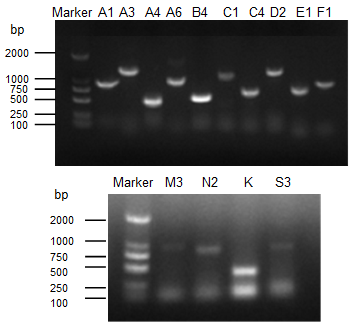
**

**Figure S4: SDS PAGE analysis of expression products of pyrimidine nucleosides functional genes from *H. Sinensis***

**
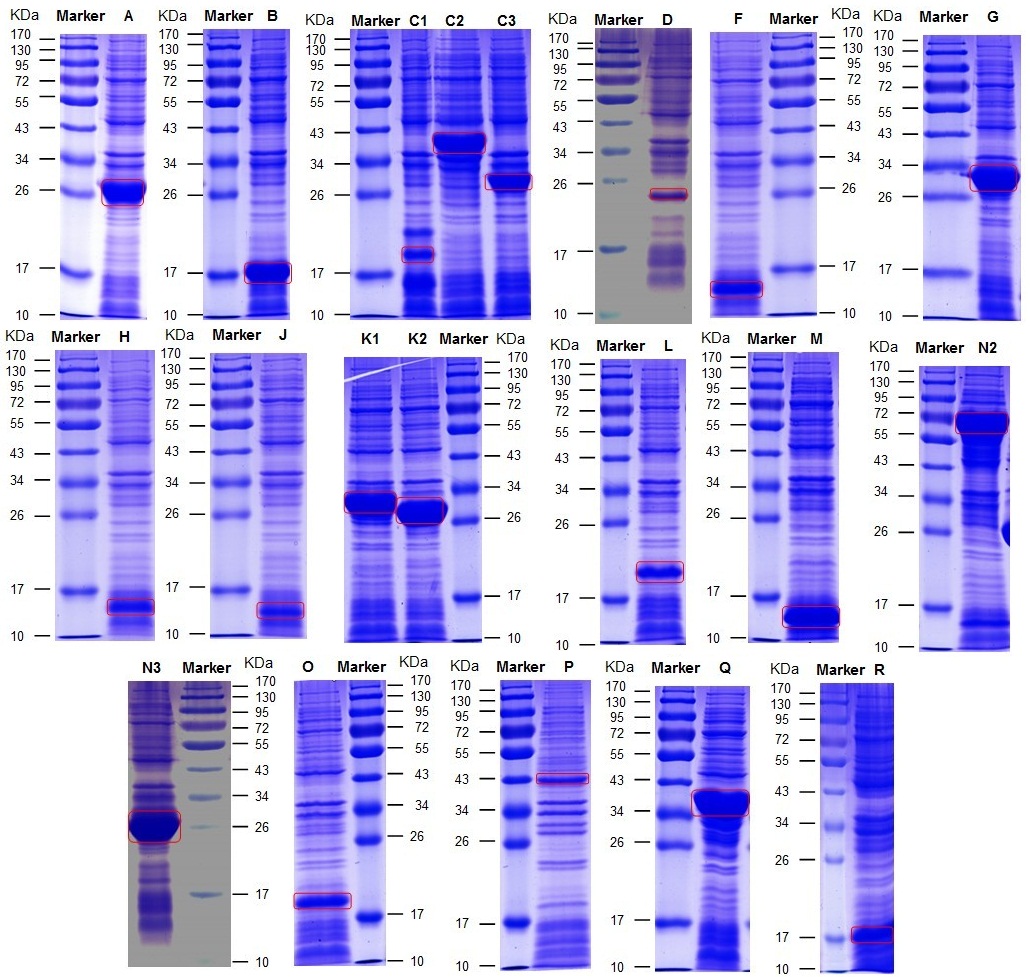
**

**Figure S5: SDS PAGE analysis of expression products of unsaturated fatty acid functional genes from *H. Sinensis***

**
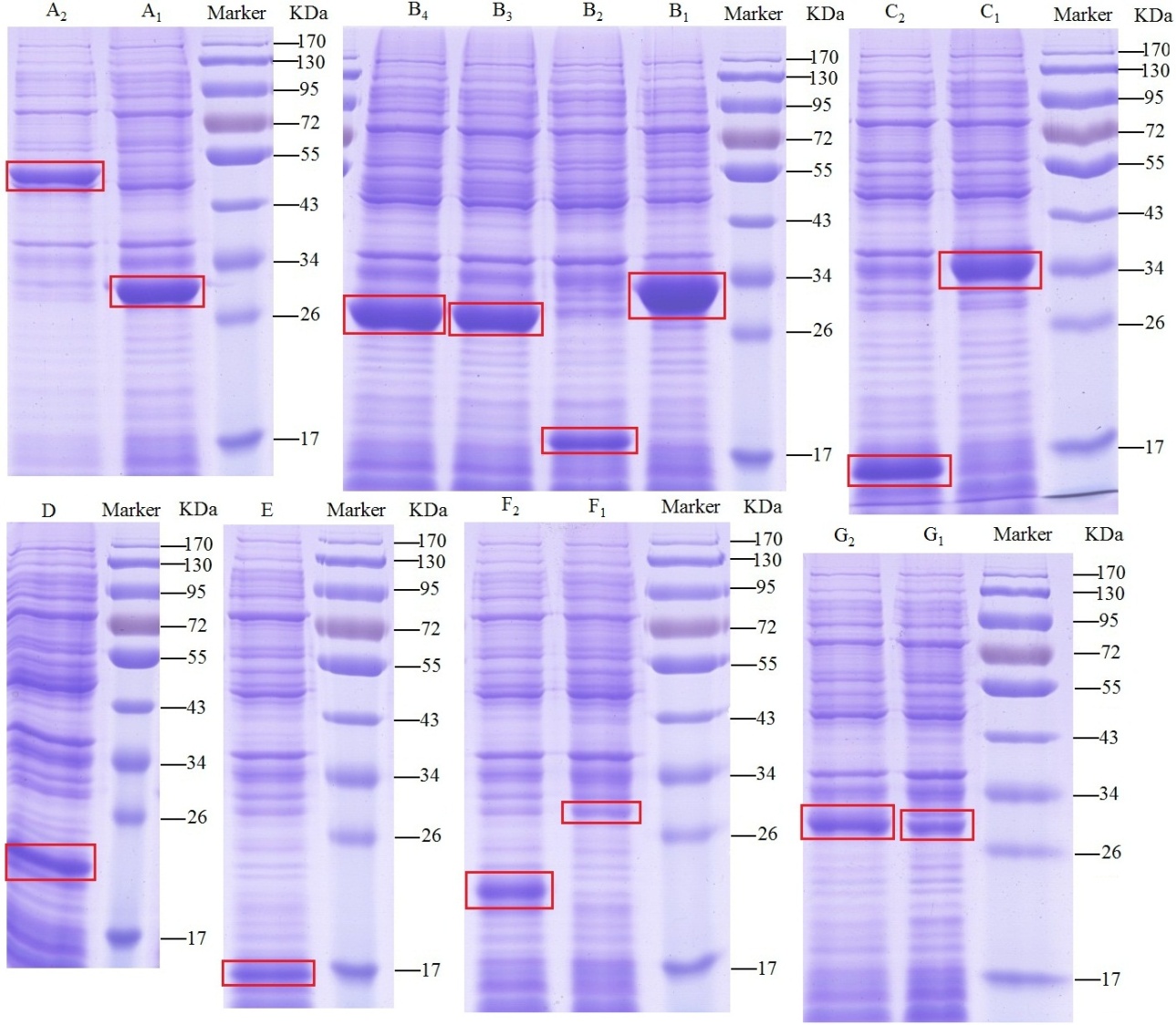
**

**Figure S6: SDS PAGE analysis of expression products of cordyceps polysaccharide functional genes from *H. Sinensis***

**
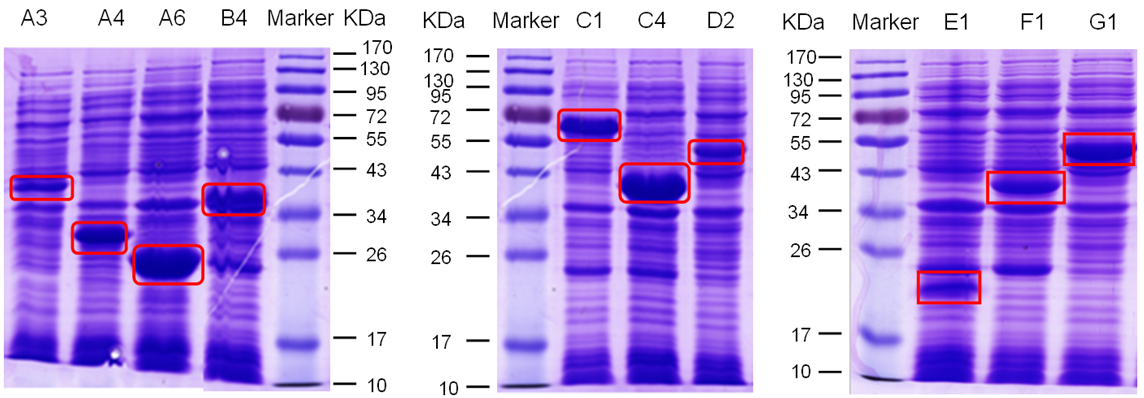
**

**Figure S7: SDS PAGE analysis of the enzymes involving in infection mechanism of *H. Sinensis***

**
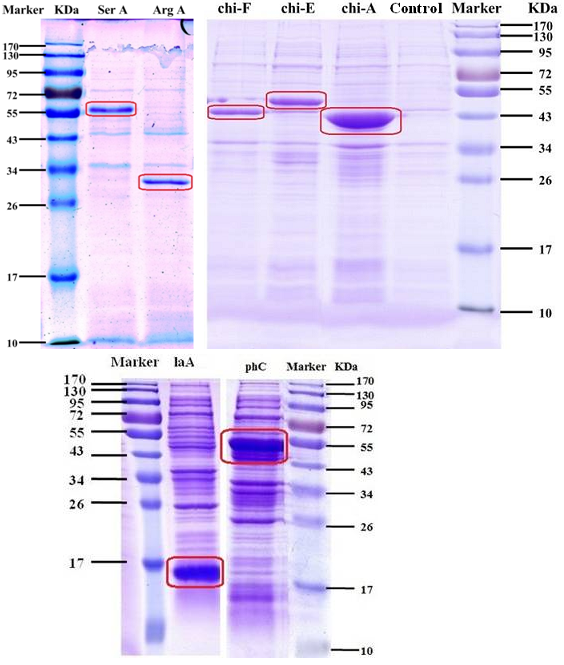
**

**Figure S8: LC-MS spectrum of cordycepin in standards and *H. Sinensis* samples. A) LC spectrum of cordycepin in standards. B) MS spectrum of cordycepin in standards. C) LC spectrum of cordycepin in *H. Sinensis* samples. D) MS spectrum of cordycepin in *H. Sinensis*samples.**


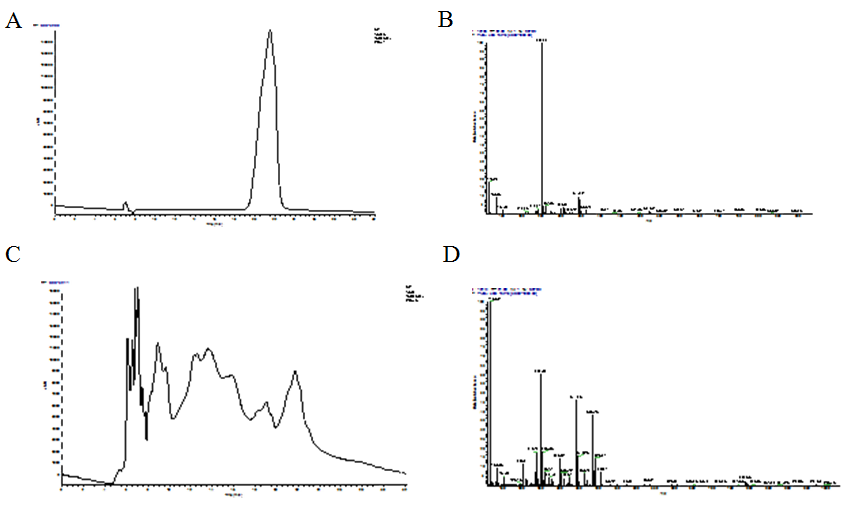


**Figure S9: Structures and MS spectrum of unsaturated fatty acid. A) Structure and MS spectrum of hexadecanoic acid. B) Structure and MS spectrum of oleic acid. C) Structure and MS spectrum of linoleic acid.**


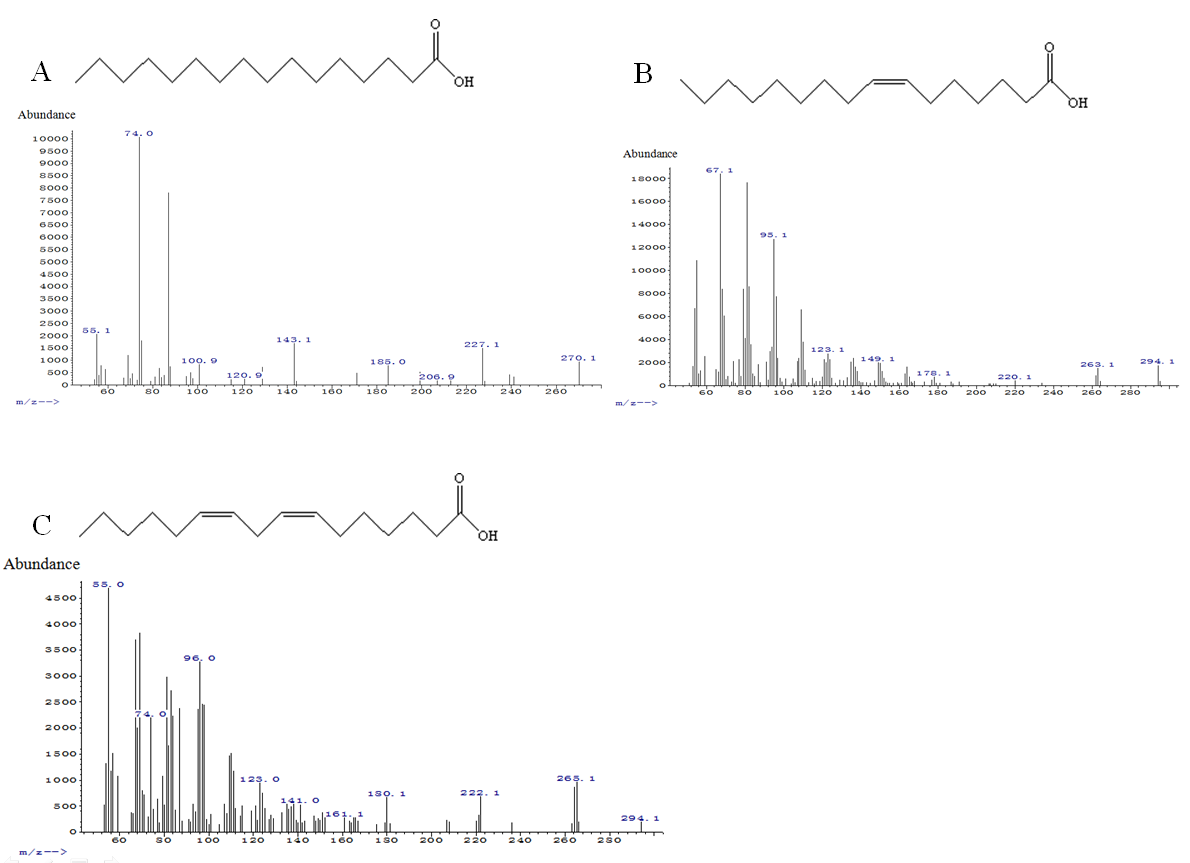


**Figure S10: Gel filtration chromatogram of polysaccharide fractions on a Sephacryl S-100 HR column. A) Gel filtration chromatogram of neutral polysaccharide for the first time. B) Gel filtration chromatogram of neutral polysaccharide fractions for the second time. The concentrated product is named as HSP-1. C) Gel-filtration chromatogram of the acidic polysaccharide for the first time. D) Gel-filtration chromatogram of the acidic polysaccharide for the second time. The concentrated product is named as HSP-2.**


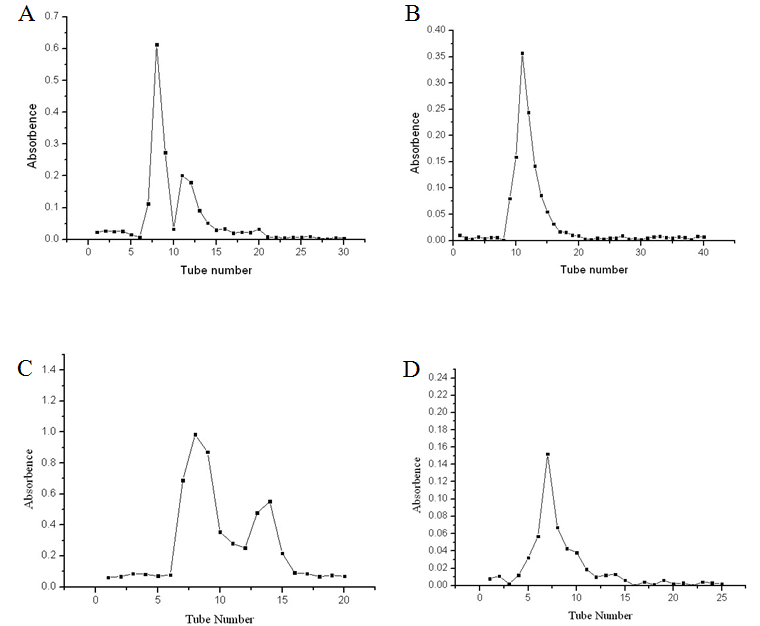


**Figure S11: GC profiles of the alditol acetates of HSP-1 and HSP-2 on GC-MS.(A)Peak identity of standards; (B) Peak identity of HSP-1:D-Man (rt:11.421min); D-Gal (rt:11.503min); D-Glc (rt:11.577min). (C) Peak identity of HSP-2: D-Man (rt:11.428min); D-Gal (11.497min); D-Glc (11.580min).**


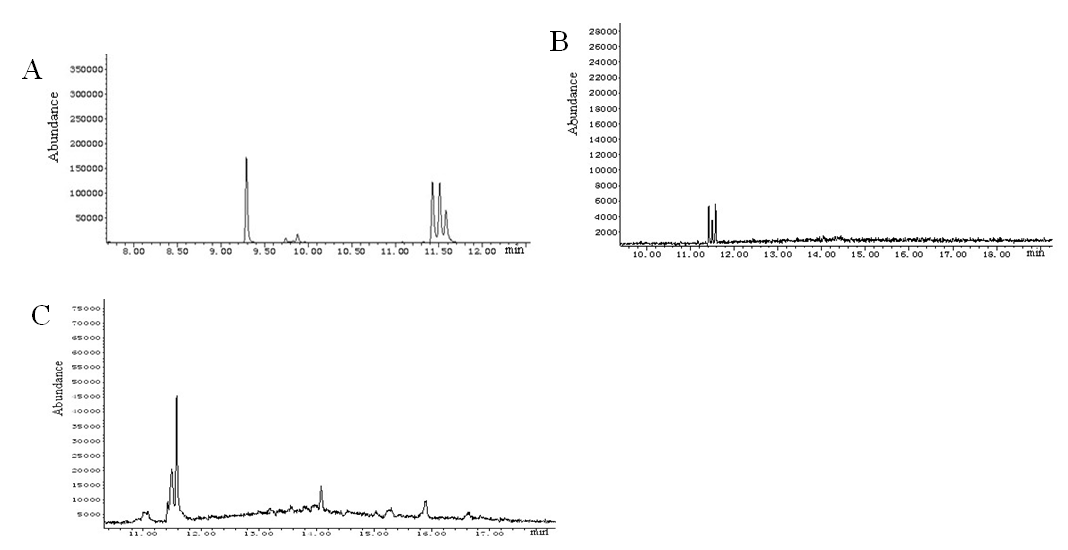


**Figure S12: IR spectrum of （A）HSP-1 and （B） HSP-2.**


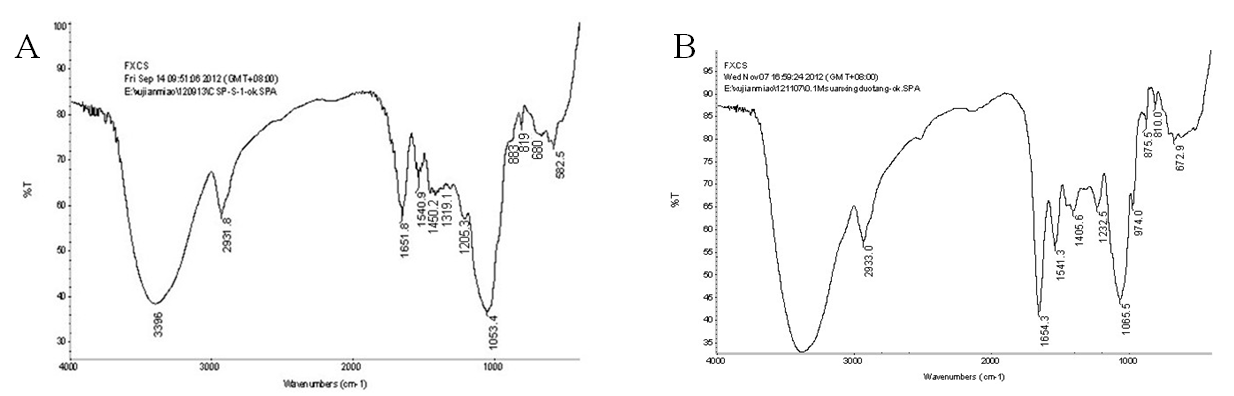


**Figure S13: ^1^H NMR (500 MHz) spectrum of （A）HSP-1 and （B） HSP-2.**


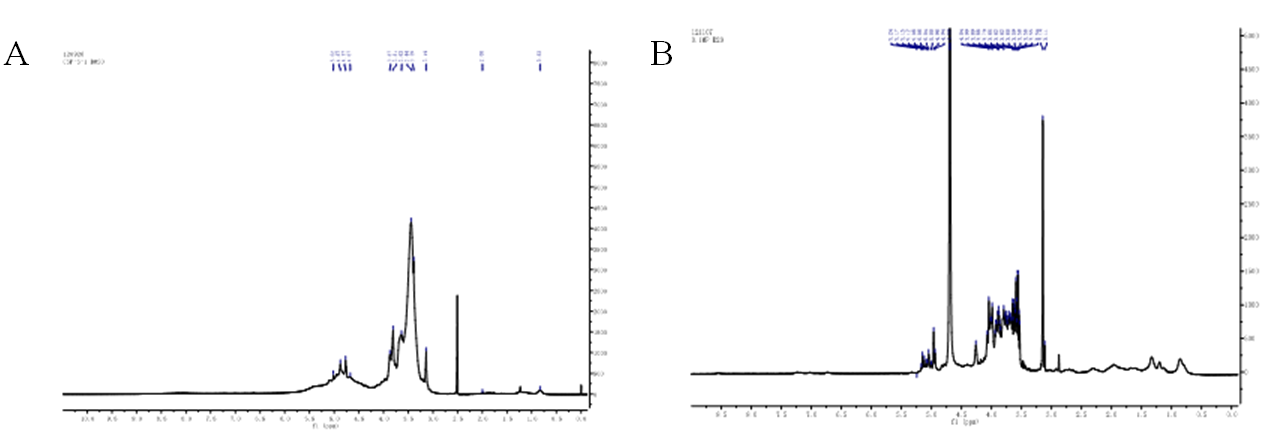


**Figure S14: ^13^C NMR (500 M) spectrum of （A）HSP-1 and （B） HSP-2.**


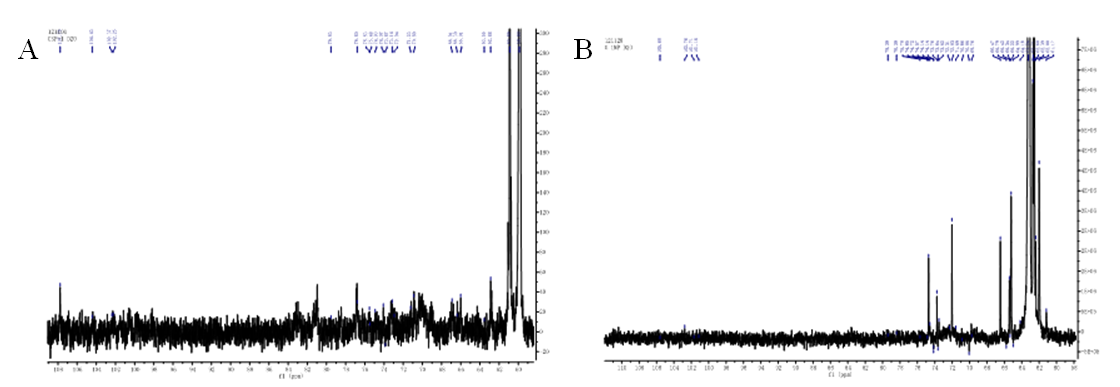


**Figure S15: SDS-PAGE of expression products of cordycepin anabolic functional genes in *H. sinensis*. Lane 1 stands for corA, lane 2 stands for corB1, lane 3 stands for corB2, lane 4 stands for corC1, lane 5 stands for corC2, lane 6 stands for corD, lane 7 stands for corE, lane 8 stands for corF, lane 9 stands for corG, lane 10 stands for corH, lane 11 stands for corI, lane 12 stands for corJ, lane 13 stands for corK, lane 14 stands for corM1, lane 15 stands for corM2 and lane 16 stands for corL.**

**Additional Tables**

**Table S1: Identification result of Biolog**

| NO. | Carbon substrate | C07-15-1 F35 | NO. | Carbon substrate | C07-15-1F35 |
| --- | --- | --- | --- | --- | --- |
| 0 | Water | − | 48 | D-Ribose | + |
| 1 | Tween 80 | − | 49 | Salicin | − |
| 2 | N-Acetyl-D-Galactosamine | − | 50 | Sedoheptulosan | − |
| 3 | N-Acetyl-ß-D-Glucosamine | − | 51 | D-Sorbitol | − |
| 4 | N-Acetyl-ß-D-Mannosamine | − | 52 | L-Sorbose | − |
| 5 | Adonitol | − | 53 | Stachyose | − |
| 6 | Amygdalin | − | 54 | Sucrose | − |
| 7 | D-Arabinose | + | 55 | D-Tagatose | − |
| 8 | L-Arabinose | + | 56 | D-Trehalose | − |
| 9 | D-Arabitol | − | 57 | Turanose | − |
| 10 | Arbutin | − | 58 | Xylitol | − |
| 11 | D-Cellobiose | − | 59 | D-Xylose | + |
| 12 | a-Cyclodextrin | − | 60 | y-Aminobutyric Acid | − |
| 13 | ß-Cyclodextrin | − | 61 | Bromosuccinic Acid | − |
| 14 | Dextrin | + | 62 | Fumaric Acid | − |
| 15 | i-Erythritol | − | 63 | ß-Hydroxybutyric Acid | − |
| 16 | D-Fructose | − | 64 | y- Hydroxybutyric Acid | − |
| 17 | L-Fucose | − | 65 | p-Hydroxy-phenylacetic Acid | − |
| 18 | D-Galactose | − | 66 | a-Ketoglutaric Acid | + |
| 19 | D-Galacturonic Acid | − | 67 | D-Lactic Acid Methyl Ester | − |
| 20 | Gentiobiose | − | 68 | L-Lactic Acid | − |
| 21 | D-Gluconic Acid | − | 69 | D-Malic Acid | − |
| 22 | D-Glucosamine | − | 70 | L-Malic Acid | − |
| 23 | a-D-Glucose | − | 71 | Quinic Acid | − |
| 24 | a-D-Glucose-1-Phosphate | − | 72 | D-Saccharic Acid | − |
| 25 | Glucuronamide | − | 73 | Sebacic Acid | − |
| 26 | D-Glucuronic Acid | − | 74 | Succinamic Acid | − |
| 27 | Glycerol | − | 75 | Succinic Acid | − |
| 28 | Glycogen | − | 76 | Succinic Acid Mono-Methyl Ester | − |
| 29 | m-Inositol | − | 77 | N-Acetyl-L-Glutamic Acid | − |
| 30 | 2-Keto-D-Gluconic Acid | − | 78 | L-Alaninamide | − |
| 31 | a-D-Lactose | − | 79 | L-Alanine | − |
| 32 | Lactulose | − | 80 | L-Alanyl-Glycine | − |
| 33 | Maltitol | − | 81 | L-Asparagine | − |
| 34 | Maltose | − | 82 | L-Aspartic Acid | − |
| 35 | Maltotriose | − | 83 | L-Glutamic Acid | − |
| 36 | D-Mannitol | − | 84 | Gycyl-L-Glutamic Acid | − |
| 37 | D-Mannose | − | 85 | L-Ornithine | − |
| 38 | D-Melezitose | − | 86 | L-Phenylalanine | − |
| 39 | D-Melibiose | − | 87 | L-Proline | + |
| 40 | a-Methyl-D-Galactoside | − | 88 | L-Pyroglutamic Acid | − |
| 41 | ß-Methyl-D- Galactoside | − | 89 | L-Serine | − |
| 42 | a-Methyl-D-Glucoside | + | 90 | L-Threonine | − |
| 43 | ß-Methyl-D-Glucoside | − | 91 | 2-Aminoethanol | + |
| 44 | Palatinose | − | 92 | Putrescine | − |
| 45 | D-Psicose | − | 93 | Adenosine | + |
| 46 | D-Raffinose | − | 94 | Uridine | − |
| 47 | L-Rhamnose | − | 95 | Adenosine-5’-Monophosphate | − |
| *Notes: +, positive; −, negative; B, borderline* | | | | | |

**Table S2: Statistics table about ncRNA**

| Type | Copy_Num | Avg_Len (bp) | Total_Len (bp) | % in Genome |
| --- | --- | --- | --- | --- |
| tRNA | 122 | 88.23 | 10765 | 0.0105 |
| rRNA (*denovo*) | 35 | 595.48 | 20842 | 0.0203 |
| sRNA | 5 | 208.2 | 1041 | 0.0010 |
| snRNA | 33 | 120.61 | 3980 | 0.0039 |
| miRNA | 0 | 0 | 0 | 0.0000 |

**Table S3: Statistics table about repeats**

| Type | Repeat Size (bp) | % in Genome |
| --- | --- | --- |
| Repbase | 7590304 | 7.3977 |
| ProMask | 18546155 | 18.0754 |
| TRF | 6252839 | 6.0941 |
| Total | 25410472 | 24.7655 |
